# Decoding the therapeutic potential of extracellular vesicles in osteoarthritis: from biomolecular composition to clinical translation

**DOI:** 10.1097/JS9.0000000000003219

**Published:** 2025-08-21

**Authors:** Wenjing Cheng, Pan Jin, Wei Liu, Ruiqi Feng, Lixue Zou, Rui Wang, Yanlong Xing, Qiong He, Juan Wang, Tongmeng Jiang

**Affiliations:** a The Joint Department of Orthopedics, The First Affiliated Hospital of Yangtze University, Jingzhou, Hubei, China; b Health Science Center, Yangtze University, Jingzhou, Hubei, China; c Key Laboratory of Emergency and Trauma of Ministry of Education, Key Laboratory of Haikou Trauma, Key Laboratory of Hainan Trauma and Disaster Rescue, The First Affiliated Hospital, Hainan Medical University, Haikou, Hainan, China; d NHC Key Laboratory of Tropical Disease Control, Engineering Research Center for Hainan Bio-Smart Materials and Bio-Medical Devices, Key Laboratory of Hainan Functional Materials and Molecular Imaging, School of Life Sciences and Medical Technology, Hainan Academy of Medical Sciences, Hainan Medical University, Haikou, Hainan, China; e Hubei Three Gorges Polytechnic, Yichang, Hubei, China; f Key Laboratory of Tropical Translational Medicine of Ministry of Education & Key Laboratory of Brain Science Research and Transformation in Tropical Environment of Hainan Province, Hainan Provincial Stem Cell Research Institute, School of Basic Medicine and Life Sciences, Hainan Medical University, Haikou, China

**Keywords:** administration, characterization, extracellular vesicles, isolation, osteoarthritis

## Abstract

Osteoarthritis (OA) is a highly prevalent degenerative joint disorder that substantially compromises the quality of life in middle-aged and elderly individuals. Conventional therapeutic approaches exhibit limited efficacy, and there is an urgent need to identify more effective treatment options. Extracellular vesicles (EVs) serve as essential mediators of intercellular communication and have been established as crucial carriers for the delivery of bioactive molecules, encompassing DNA, RNA species (including mRNAs, lncRNAs, microRNAs), proteins, and lipids, in the pathogenesis and repair of OA. Comprehensive research has demonstrated that EVs derived from diverse sources possess significant therapeutic potential in mitigating OA progression. However, their dual role in simultaneously facilitating the transport of both beneficial and harmful factors necessitates a cautious interpretation. This review aims to systematically investigate the roles of EVs derived from various origins and subpopulations in mitigating OA progression, summarize recent advancements in EV delivery methodologies, and emphasize emerging strategies to enhance their therapeutic specificity and efficacy. By elucidating these mechanisms, this review seeks to address translational challenges and provide valuable insights into the development of next-generation EV-based therapeutics for OA treatment.

## Introduction

Osteoarthritis (OA) is a prevalent degenerative joint disease among middle-aged and elderly individuals, primarily impacting the knee, hip, and hand joints. Notably, knee OA accounts for approximately 7% of the global disease burden^[1]^. The pathophysiology is marked by irreversible chondrocyte loss and abnormal osteogenic differentiation, with histological features including cartilage defects and terminal joint bone hyperplasia^[2]^. Clinical manifestations evolve from initial inflammatory symptoms and mobility restrictions to advanced structural changes, such as osteophyte formation, joint space narrowing, and eventual deformity^[3,4]^. Despite extensive research efforts, effective interventions for the restoration of hyaline cartilage remain limited and difficult to achieve^[5,6]^. Cartilage regenerative failure can be attributed to the limited cellular renewal capacity, biomechanical abnormalities, inflammatory microenvironments, and metabolic dysfunction^[7,8]^. Given that articular cartilage consists of chondrocytes embedded within the extracellular matrix (ECM), the reduction in chondrocyte number or function represents a critical factor in the pathogenesis of OA^[9,10]^. Although the mechanisms underlying chondrocyte depletion remain incompletely understood, potential contributors may involve apoptotic processes, aberrant differentiation pathways, and impaired progenitor cell differentiation^[11,12]^. Clinical investigations have shown that intra-articular stem cell administration alleviates symptomatic manifestations and improves joint functionality. However, the determination of long-term efficacy remains to be further evaluated^[13–16]^. Despite preliminary evidence of efficacy, concerns regarding immunogenicity, oncogenic potential, and ethical considerations remain unresolved^[17]^.

**HIGHLIGHTS**

- Extracellular vesicles (EVs) are pivotal carriers for delivering bioactive molecule in osteoarthritis pathogenesis and repair.
- There are both beneficial and harmful factors warrants cautious interpretation of EVs.
- Emerging strategies to optimize the therapeutic specificity and efficacy of EVs are discussed.

**Figure 1.**
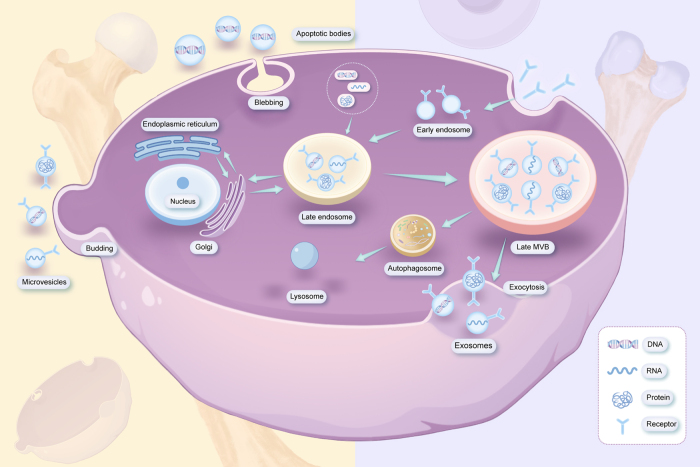
Schematic illustration of EV secretion: exosomes, microvesicles, and apoptotic bodies.

Extracellular vesicles (EVs), which function as mediators of intercellular communication, have attracted significant research attention as potential alternative therapeutic agents. Compared with cellular interventions, EVs avoid immunological rejection and demonstrate enhanced stability, storage efficiency, and manufacturability, with multiple studies indicating potentially superior therapeutic efficacy compared to stem cell therapies^[18,19]^. EVs are membrane-bound structures ubiquitously present in biological fluids and cellular supernatants, capable of facilitating the stable transport of diverse signaling molecules. Based on their biogenesis mechanisms and dimensional characteristics, EVs are categorized into three primary classifications: exosomes (30-150 nm in diameter), microvesicles (150-1000 nm in diameter), and apoptotic bodies (>1000 nm in diameter) (Table 1). Exosome formation involves endocytosis leading to the generation of early endosomes, subsequent inward budding resulting in multivesicular body formation, and eventual plasma membrane fusion for exosome release. Microvesicles arise through direct plasma membrane budding, whereas apoptotic bodies are formed during cellular apoptosis^[20,21]^ (Fig. 1). Despite biogenetic and dimensional differences between exosomes and microvesicles, both harbor parental nucleic acids and proteins with overlapping size distributions, thereby complicating their complete isolation^[22]^. Given their distinct origins and compositional profiles, EVs exhibit substantial immunomodulatory functions^[23]^. In inflammatory contexts, immune cell-derived EVs regulate systemic responses. Notably, neutrophil-derived EVs are internalized by macro-phages, thereby inhibiting the progression of inflammation^[24]^. EVs transfer anti-inflammatory mediators and suppress inflammatory cytokine release, as exemplified by apoptotic extracellular vesicles inhibiting TNF-α secretion^[25]^. Additionally, EVs modulate immune cell phenotypes by promoting M2 macrophage polarization and regulatory T-cell differentiation, thereby mediating anti-inflammatory and reparative effects^[26–29]^. The composition of EVs is modulated by specific pathophysiological conditions. Indeed, inflammatory responses and tissue damage can trigger increased EV release^[30]^. These vesicular structures mitigate OA progression through the following mechanisms: (1) inflammatory modulation achieved by targeted delivery of anti-inflammatory factors and inhibition of signaling pathways; (2) enhancement of chondrocyte proliferation and inhibition of apoptosis via transport of growth factors, activation of signaling cascades, and regulation of autophagy, mitochondrial function, and ferroptosis; and (3) regulation of ECM metabolism by promoting synthesis and inhibiting degradation^[31–33]^. Compared with conventional monotherapeutic OA interventions, EVs show greater promise for application in cartilage repair. Combined EV-hyaluronic acid therapy demonstrates significantly enhanced efficacy compared with hyaluronic acid monotherapy^[34,35]^. While intra-articular EVs may exhibit bidirectional inflammatory modulation, co-administration of hydrogel mitigates pro-inflammatory risk, thereby establishing novel therapeutic strategies for OA management^[36]^.

**Table 1:** Differences among exosomes, microvesicles and apoptotic bodies

Classification	Exosomes	Microvesicles	Apoptotic bodies
Diameter	30–150 nm	150–1000 nm	>1000 nm
Density	1.11–1.19 g/mL	1.02–1.22 g/mL	1.16–1.28 g/mL
Source	The multivesicular bodies within the cell fuse with the plasma membrane and are subsequently released extracellularly	Cell membrane protrusion and budding	Release during apoptosis
Protein marker	Alix, TSG101, HSC70, CD63, CD81, CD9	Selectin, Integrins, CD40, MMP	Histones
Key Cargo	miRNAs, proteins, lipids. High specificity.	mRNAs, proteins, lipids, organelles (e.g., mitochondria).	DNA fragments, organelles, cellular debris.
Primary Therapeutic Strength	Potent signaling, tissue penetration, low immunogenicity, targeted anti-inflammation.	Robust regenerative capacity, mitochondrial transfer, superior cargo loading.	Powerful immunomodulation (via phagocytosis), clearance of inflammation.
Clinical Limitations	Low production yield, complex purification.	Potential for pro-inflammatory effects, thrombogenicity risk, purification challenges.	Risk of inducing autoimmune responses, limited cell sources, purification challenges.

The therapeutic potential of EVs has recently emerged as a promising frontier in the management of OA. While existing literature has provided valuable insights, previous reviews have predominantly focused on a single vesicle subtype – the exosome – or have adopted a broad perspective on EV applications across various diseases^[37]^. Others have centered on specific mechanisms, such as EV-extracellular matrix interactions within biomaterials^[38]^. However, the therapeutic promise of EVs extends beyond exosomes alone. This review addresses a critical gap by providing a systematic and comparative analysis of all three primary EV classes: exosomes, microvesicles, and apoptotic bodies, along with their potential to mitigate OA progression. We explore the entire translational continuum – from biomolecular cargo and isolation techniques to bioengineering and delivery strategies – highlighting the unique strengths, challenges, and clinical applicability of each class. Thus, this work not only complements existing literature but also establishes a foundational framework and strategic roadmap, guiding future research toward the development of safer, more potent, and precisely targeted next-generation cell-free therapeutics for OA.

## Isolation and characterization of EVs

EVs serve as key mediators in intercellular communication, molecular transport, and the processes of tissue repair and regeneration. To enhance the feasibility of both research and clinical applications, it is essential to isolate these vesicles into distinct subpopulations. This section examines advanced methodologies utilized for EV isolation and characterization.

### Isolation of EVs

EV isolation methodologies encompass a range of approaches (Table 2), with ultracentrifugation (UC) being the most widely utilized technique^[39,40]^. However, due to the overlapping physicochemical properties between EVs and non-vesicular extracellular particles (NVEPs), such as protein aggregates and lipoprotein complexes, no current method can guarantee complete EV separation. For isolating EVs from cell culture media, human urine, and plasma, precipitation-based protocols generate preparations with lower purity, whereas ultracentrifugation combined with size exclusion chromatography and ultrafiltration demonstrates matrix-dependent efficacy^[41]^. Each isolation strategy offers specific advantages and limitations. Notably, Reimer *et al* compared UC with ultrafiltration (UF) for outer membrane vesicle isolation from Gram-negative bacteria. Nanoparticle tracking analysis (NTA) demonstrated that UF achieved higher vesicle yield, enhanced separation efficiency, and improved capacity for isolating smaller-sized vesicles^[42]^. Additionally, microfluidic technologies, characterized by cost-effectiveness and high throughput, have been widely implemented in EV isolation^[43]^. Li *et al* developed a cascaded microfluidic pulsatile filtration system that enables the efficient separation of high-purity, high-yield EVs from blood within 30 minutes, offering a significantly more time-efficient alternative to UC^[44]^. Each separation method has its unique advantages. According to research results, although UC is complex and time-consuming, it remains the first choice when higher purity EVs are required for experiments. However, for applications that pursue high efficiency and large-scale production, UF and qEV column chromatography (based on the principle of size-exclusion chromatography) offer faster separation speeds and higher particle concentrations^[45]^.

**Table 2:** The commonly employed methods for size-based separation of extracellular vesicles (EVs)

Separation techniques	Principle	Advantages	Limitations	Clinical applicability	Citations
Ultracentrifugation	By leveraging the distinct physical properties of EVs, specifically their density and size variations relative to other cellular constituents	This technique is characterized by its simple operation, low cost, and the capacity for large-volume samples	This technique demonstrates relatively low separation accuracy, reduced processing efficiency, and requires sophisticated instrumentation	It is the most commonly used EVs purification method, including density gradient ultracentrifugation and differential ultracentrifugation. Its unstable purity, high labor intensity, and difficulty in processing multiple biological samples within a short time frame limit its large-scale clinical application.	^[39-42,46-49]^
Size-exclusion chromatography	By utilizing porous gel packing materials, macromolecules are eluted preferentially, while smaller molecules exhibit retarded elution profiles	This technique utilizes mild separation conditions, thereby preserving the structural integrity and bioactivity of EVs	This technique is characterized by a slow processing speed and limited sample throughput	Compounds with similar molecular sizes cannot be effectively distinguished. Significant separation is only achievable when the difference in molecular weight exceeds 10%. The research and development of chromatographic columns with specific pore sizes for constructing molecular sieve systems remains a key factor limiting their large-scale production and clinical application.	^[41,46,47,50]^
Ultrafiltration	By utilizing ultrafiltration membranes with defined pore sizes, components smaller than the pore diameter can pass through the membrane, while larger components are effectively retained	This technique is characterized by its straightforward and rapid operation, making it particularly suitable for small-scale sample processing	This technique is associated with inadequate impurity removal and significant loss of EVs	The presence of a large number of non-EV nanovesicles similar in size to EVs compromises the purity of separation. This scheme is relatively well-suited for large-scale production and clinical application. However, the adsorption of extracellular vesicles onto the filter membrane may shorten its lifespan and compromise the performance of the filter membrane, potentially hindering its clinical promotion.	^[41,42,51]^
Microfluidic technologies	By leveraging precisely engineered microchannel configurations and hydrodynamic mechanisms in microfluidic devices, efficient size-based separation of EVs can be achieved	This technique is characterized by fast operation, excellent accuracy, and requires only small sample volumes	This technique requires advanced operational skills and incurs high implementation costs	A comparison of physical and chemical microfluidic methods for EV separation reveals a slight advantage of chemical methods over physical ones. The physical microfluidic method demonstrates higher working efficiency, whereas the chemical microfluidic method exhibits superior performance. Striking a balance between these two approaches represents a critical factor influencing clinical application.	^[43,44,52]^
ExoEasy membrane affinity method	Separation is performed using membranes modified with affinity ligands as the medium	The operation process is relatively straightforward, and it can efficiently separate high-purity EVs with minimal damage to the samples.	High cost, expensive adsorbents, and low separation efficiency are significant limitations of this method.	Owing to its simple operation and high purity, this method is suitable for small-scale usage, such as in laboratories, but may not be appropriate for large-scale production or clinical applications.	^[53]^
Polymer-based purification	By hijacking the water molecules surrounding EVs to generate a hydrophobic microenvironment, EVs are compelled to leave the solution and precipitate during low-speed centrifugation	This method boasts simplicity, scalability, high yield, and the ability to preserve EV integrity, enabling processing of a large number of samples without requiring additional equipment.	The purity of the obtained EVs is relatively low, with a high content of impurity proteins.	The heterogeneity of EVs is a key factor limiting their clinical efficacy, while the low purity of EVs extracted using this protocol significantly hinders their clinical application.	^[54]^
Magnetic bead-based/affinity methods	Magnetic beads coated with marker antibodies are incubated with EVs. Following antibody binding to the EV, the EVs are adsorbed and separated, enabling the capture of different types of EVs from the sample.	EVs isolated using immunoprecipitation technology exhibit high specificity, involve simple operation, and maintain the integrity of EV morphology.	The low separation efficiency, along with the susceptibility of EV biological activity to pH and salt concentration, renders this method unfavorable for downstream experiments.	It is only applicable to small-sample studies, as more expensive antibodies are required for large-sample issues, thereby limiting its large-scale application and clinical use to some extent.	^[55]^
ExoQuick method	Precipitating EVs using polyethylene glycol (PEG)	The simplicity of operation and low cost make this method an optimal choice for researchers dealing with small sample sizes, such as clinical research samples or small animal models.	Compared with other extraction schemes, the purity and quality of the EVs extracted by this method need further in-depth research.	Designed for laboratory use, not suitable for clinical use.	^[56]^

Multiple studies have consistently shown that the integration of complementary isolation methodologies not only substantially increases the yield of EVs but also markedly improves their purity profiles^[57,58]^. Visan *et al* demonstrated that tangential flow filtration (TFF) combined with size exclusion chromatography (SEC) achieves superior vesicular recovery rates and enhanced cost-efficiency compared to UC for the isolation of small EVs (sEVs)^[46]^. Comparative analyses conducted by Grenhas *et al* between sucrose cushion ultracentrifugation (sUC) and ultrafiltration coupled with size exclusion chromatography (UF-SEC) demonstrated the superior operational performance and fractionation efficiency of UF-SEC^[47]^. Additionally, minimally invasive methodologies, such as sequential filtration, exhibit significant utility in the processing of high-volume biofluids (e.g., urine, conditioned media). However, these approaches are typically associated with reduced efficiency coefficients and increased operational costs^[59]^. As research advances, the strengths and limitations of various separation methods become increasingly evident. Selecting an appropriate electrophoresis separation method according to the experimental requirements is likely to be a key consideration for future related studies^[60]^.

The existing literature primarily focuses on cell lines or liquid biofluids, leaving tissue-derived vesicular preparations largely under-characterized. To address this methodological gap, Crescitelli *et al* developed a standardized protocol enabling the isolation and characterization of six distinct EV subpopulations from neoplastic tissue matrices, including murine colorectal carcinoma and human melanoma specimens^[61]^. Martínez-Greene *et al* developed an optimized hybrid methodology that combines ultracentrifugation with iodixanol-sucrose density gradient fractionation for efficient EV isolation from murine hepatic and adipose tissues. Subsequently, they validated vesicular integrity using immunoblot analyses and transmission electron microscopy (TEM)^[48]^. Recent technological advancements have led to the development of novel size-selective EV isolation platforms. Notably, Weerakkody *et al* engineered a photolabile lipid nanoprobe that facilitates the dimensional discrimination of EVs within complex biological matrices. The controlled photodegradation of the probe moiety enables the subsequent release of vesicles while preserving their structural and functional integrity, closely approximating native conformational states^[62]^. By using resistive pulse sensing (RPS) technology and relying on the precise performance of NanoCoulter, Yang M *et al* conducted a comprehensive evaluation of five extracellular vesicle separation methods. It was found that although ultrafiltration, qEV column chromatography, and ExoQuick methods could achieve higher particle concentrations, the traditional differential ultracentrifugation method performed best in terms of purity^[45]^. With the growing attention on exosomes, novel extraction techniques continue to emerge. In comparative analyses of the strengths and limitations of existing extraction methods, identifying a more suitable technique for clinical translation will be the primary focus of future research.

### Characterization of EVs

Following isolation, the comprehensive characterization of distinct EV subpopulations represents an essential analytical requirement. Systematic identification and classification of EV subsets depend on multiparametric analysis of their biophysical properties (e.g., hydrodynamic diameter, buoyant density), biochemical composition (e.g., transmembrane and cytosolic proteins, nucleic acids, lipid constituents), and functional biological attributes within both physiological and pathophysiological contexts. EV characterization methodologies encompass both physical and biochemical analytical approaches^[63]^. Prevalent physical characterization techniques include particle dimensional analysis (e.g., nanoparticle tracking analysis, dynamic light scattering) and ultrastructural morphological assessment (e.g., transmission electron microscopy, scanning electron microscopy). By leveraging the principles of light scattering, NTA quantifies Brownian motion parameters to determine vesicular size distributions and concentration metrics. This methodology has gained widespread adoption owing to its relative simplicity and ease of implementation. Given the limited discriminatory capacity of conventional scattering-mode NTA for particles with dimensions similar to exosomes, fluorescence-mode NTA (FL-NTA) has been developed as an advanced analytical platform. FL-NTA utilizes fluorophore-conjugated markers targeting EV-specific tetraspanin proteins (e.g., CD9, CD63, CD81) to enable rapid quantification of vesicular dimensions, concentration, and subpopulation differentiation^[64]^, although it is subject to inherent methodological limitations. Magdalena Dlugolecka *et al* systematically assessed the performance of FL-NTA across EVs derived from diverse biological sources, demonstrating that optimal analytical performance requires high sample purity, while suboptimal efficacy is observed for vesicular preparations derived from plasma^[65]^. Multiple investigations have shown that NTA exhibits reduced analytical sensitivity for smaller EVs (<70 nm), whereas SEM demonstrates superior detection capacity for these vesicular subpopulations, providing analytical advantages for small EV characterization^[66]^. Morphological assessment methodologies remain extensively utilized. For instance, TEM and SEM analyses of sea cucumber-derived EVs revealed spherical sEVs (60–480 nm) with enriched protein constituents, which were subsequently demonstrated to possess anti-inflammatory functional properties^[67]^.

Biochemical characterization methodologies primarily include protein profiling (immunoblot analysis, enzyme-linked immunosorbent assay [ELISA]), nucleic acid analysis (high-throughput sequencing, reverse transcription-polymerase chain reaction [RT-PCR]), and lipid composition evaluation (thin-layer chromatography [TLC], liquid chromatography). Among these analytical approaches, immunoblotting stands out as the most widely adopted and methodologically advanced technique for detecting extracellular vesicle markers. Raman spectroscopy, an advanced single-particle optical imaging (OSPI) technique, exhibits significantly improved analytical sensitivity and precision compared to conventional characterization methods^[68]^. To enhance the feasibility of both research and clinical applications, the precise isolation of EVs into distinct subpopulations is essential. Although numerous isolation techniques have been developed, ultracentrifugation (UC) remains the most widely used method^[39,40]^. However, current methodologies exhibit limited absolute specificity because of the overlapping physicochemical properties between EVs and non-vesicular extracellular particles (NVEPs), such as protein aggregates and lipoprotein particles. Emerging microfluidic platforms show significantly higher efficiency in EV isolation and characterization compared to conventional techniques. Nonetheless, challenges remain regarding manufacturing complexity and scalability^[43,44,69]^. The heterogeneity of EVs further complicates their comprehensive characterization. The MISEV2023 guidelines propose nine standardized characterization strategies, including analyses based on physical properties (e.g., nanoparticle tracking analysis for size profiling), morphological visualization (e.g., electron microscopy), and biochemical assessments (e.g., western blotting for marker detection)^[70]^. Recent research also highlights the application of fluorescence tags for EV labeling^[71]^. Multi-modal analytical approaches have become a standard practice to address this complexity. For instance, recent studies suggest that mesenchymal stem cell-derived EVs (MSC-EVs) should be rigorously validated through the integration of morphological observations with dual verification of canonical markers (CD9, CD63, CD81) and cell-specific surface proteins^[72]^.

Pathophysiological conditions induce alterations in EV marker expression profiles, thereby facilitating the mechanistic elucidation of disease pathogenesis. Synovial fluid (SF) contains abundant exosomal populations whose physicochemical attributes may play critical roles in the initiation and progression of OA^[73]^. Zhang *et al* utilized high-resolution flow cytometric analysis combined with surface epitope immunophenotyping to identify pathogenically relevant SF EV markers, including chondroitin sulfate proteoglycan 4 (CSPG4), CD109, biglycan (BGN), and neuropilin-1 (NRP1). Notably, CSPG4 and CD109 demonstrated elevated expression in damaged cartilage chondrocytes, while NRP1 was predominantly localized to synovial immunocytes. SF specimens of OA exhibited significant upregulation of CSPG4+ and VSIG4+ EV subpopulations, indicating these disease-associated vesicular phenotypes as potential diagnostic biomarkers and therapeutic targets^[74]^. Owing to procedural complexities and potential infectious complications associated with SF acquisition, blood-derived EV analysis represents a more practical alternative. Investigative studies have shown that platelet-poor plasma-derived EV (PPP-EV) markers aid in early OA diagnosis. Comparative MAGPix immunoassay analysis of PPP-EVs isolated from healthy controls versus early- and late-stage OA cohorts confirmed significant correlations between the expression levels of CD45, CD326, and CD56 and the pathogenesis of early OA^[75]^. Zhang *et al* identified pathogenic plasma-derived EV populations that are predictive of radiographic OA progression, particularly large vesicular subsets carrying fibrinogen alpha chain (FGA), fibrinogen beta chain (FGB), and talin (TLN) markers^[76]^. Consequently, the selective isolation of pathogenic EV subpopulations based on surface immunophenotyping – exemplified by immunomodulatory vesicles that attenuate inflammatory cascades – may represent novel therapeutic strategies for OA management. Additionally, the detection of EVs exhibiting differential marker expression profiles may facilitate the development of improved clinical OA staging algorithms.

### Technical challenges in resolving EV heterogeneity

The heterogeneity of EVs refers to the phenomenon where individual vesicles within a population do not uniformly possess all the chemical or physical properties observed in the entire population. In other words, while the exosome population as a whole may display specific functional capabilities, individual vesicles within that population may not necessarily possess those functions. This phenomenon can primarily be attributed to the diversity of cell types within the body, where different cells secrete distinct types of EVs. Moreover, even the same cell type can secrete EVs with varying characteristics. EV heterogeneity significantly impacts their diagnostic and therapeutic applications, thus underscoring the pressing need for in-depth investigation into this area. Based on whether the source cells are identical, the heterogeneity of EVs can be categorized into intra-subpopulation heterogeneity and inter-subpopulation heterogeneity^[77]^. On one hand, intra-subpopulation heterogeneity encompasses the variation in diameter, composition, and function of EVs secreted by the same cell type. On the other hand, inter-subpopulation heterogeneity encompasses the structural, compositional, and functional variations among EVs derived from different cell sources and types. Intra-subpopulation heterogeneity is primarily manifested in physical characteristics such as diameter and density, which result from the same cell source. Nanoparticle tracking analysis (NTA) was frequently employed to statistically analyze the particle size and concentration of the extracted exosomes, as well as perform a preliminary quality assessment. This device can determine the particle size distribution of particle ensembles but is unable to accurately measure the diameter of individual particles. Furthermore, it lacks the ability to distinguish among particles, contaminants, and EVs in solution. In a comparative study, Bachurski *et al* evaluated the accuracy and repeatability of the NanoSight NS300 and ZetaView. They found that ZetaView offers more accurate and reproducible descriptions of EV concentration, whereas the NanoSight NS300 provides higher-resolution size measurements. However, a key finding was that neither device is capable of reporting peak EV diameters below 60 nm^[78]^. Auger *et al* identified an additional limitation of NTA when analyzing extracellular vesicles: its inability to detect a decrease in EV secretion, although it can identify an increase^[79]^. Although NTA can detect the presence of certain contaminants in some models through fluorescence mode, it has inherent limitations due to the low scattering efficiency of biological nanoparticles. For most EVs, the actual detection limit of NTA is approximately 70 to 90 nanometers^[77]^. When the concentration is low, NTA can perform the detection task satisfactorily, whereas dynamic light scattering (DLS) is more suitable for detecting samples with higher concentrations. DLS, photon correlation spectroscopy (PCS) or quasi-elastic light scattering (QELS), offers better cost-effectiveness in particle size analysis. It is suitable for the detection of high-concentration samples, but if the samples aggregate, its detection results will be greatly distorted. Therefore, it is necessary to carry out dispersion treatment on the samples, such as ultrasonic treatment, before conducting the detection^[80]^. Compared with NTA and DLS, resistive pulse sensing (RPS) has higher sensitivity and resolution in measuring the diameter of particles. The detection limit of RPS is usually associated with the size of micropores and the noise level in the sample. If the background noise is low, RPS can detect very small and low-concentration particles, thereby achieving a lower detection limit^[70]^. As an upgraded version of DLS, multi-angle light scattering (MALS) can provide detailed information on particle size distribution, molecular weight and particle interactions by simultaneously measuring scattered light at multiple angles. MALS assesses the quality and stability of proteins by analyzing the size and distribution of protein aggregates, but it is not as effective as NTA in measuring the particle size distribution^[81]^. Raman spectroscopy (RS) is capable of providing molecular fingerprint spectra of EVs. When integrated with light scattering technology, it can concurrently measure their size. However, the technique remains limited by the low refractive index and weak scattering properties of EVs^[77]^. Scanning electron microscopy (SEM) is a commonly used imaging tool for obtaining the microstructure of a sample’s surface. Compared with SEM that forms an image by scanning the sample surface with an electron beam, transmission electron microscopy (TEM) forms an image by using an electron beam to penetrate the sample. Conventional electron microscopy faces significant challenges when imaging biological samples. For instance, water molecules vaporize during sample preparation, causing cell rupture. Additionally, excessive electron irradiation can destroy protein structures. To overcome these limitations, scientists developed cryo-electron microscopy (Cryo-EM). This technique involves rapidly freezing biological samples, which allows for their observation under an electron microscope while preserving their native state. However, this equipment is extremely expensive and has high requirements for sample preparation techniques. At the same time, when the similarity of different conformations in the sample is too high, it is very difficult to distinguish them^[70]^. Scanning probe microscopy (SPM), including scanning tunneling microscopy (STM) and atomic force microscopy (AFM), is the first microscope that is widely recognized as having atomic resolution capability. STM acquires surface topography by measuring the variation of tunneling current as a function of distance, while AFM achieves this by detecting the force-distance curve^[82]^. The images observed by SPM can more directly reflect the original characteristics of the sample, but they cannot effectively reflect the properties of the sample. From the conventional optical microscope to the diffraction-limited fluorescence microscopy and the interference reflection imaging microscope, this represents a continuous improvement in microscopic imaging accuracy achieved through the incorporation of diffraction and interference reflection principles. Ultimately, super-resolution technology has successfully overcome the diffraction limit of optical imaging through the application of various principles. The resolution of the three types of microscopes has been progressively improving, yet each still possesses its unique advantages and limitations. The diffraction-limited fluorescence microscope is characterized by high sensitivity, the interferometric reflectance imaging sensing (IRIS) exhibits high contrast, and the super-resolution microscopy (SRM) overcomes the diffraction limit. However, the diffraction-limited fluorescence microscope is prone to background interference, the IRIS microscope imposes high demands on sample preparation, and SRM technology involves significant complexity^[83]^. Bead-based flow cytometry, single-EV flow cytometry and high-resolution flow cytometry represent distinct branches of flow technology. Their core differences reside in the detection targets and technical objectives, yet they all share the fundamental principles of flow cytometry, such as liquid flow focusing, laser excitation, and optical signal detection. Single-EV flow cytometry integrates single-EV technology with conventional flow cytometry. Microbead flow cytometry extends the detection capability to soluble molecules^[84]^. High-resolution flow cytometry addresses the bottleneck of multi-color experiments via advanced optical systems and algorithmic improvements^[85]^. Together, these three approaches form a technological advancement chain^[84,85]^. Methods such as western blotting (WB) and enzyme-linked immunosorbent assay (ELISA), which are grounded in the principle of antigen-antibody binding, continue to play a crucial role in the identification and characterization of extracellular vesicles. In addition, the strengths and limitations of various methods, including genetic protein tagging, mass spectrometry proteomics, nucleic acid characterization, protein- and non-protein labelling of EVs, droplet microfluidic, biosensing, and single-vesicle technologies are summarized in Table 3. The primary methods for identifying the heterogeneity of EVs encompass: identification based on physical characteristics (e.g., NTA, DLS, RPS, SEM, TEM, etc.), identification based on surface markers (e.g., flow cytometry, WB, protein- and non-protein labeling, etc.), and emerging technologies (e.g., droplet microfluidic, RS, Biosensing, etc.). Reducing the heterogeneity of EVs remains a bottleneck issue and an active research focus. To date, no single method has been developed that can comprehensively and accurately characterize EV heterogeneity. The analysis of EV heterogeneity requires the integration of multiple techniques, such as the “triple identification method” recommended by ISEV (Western blot markers for protein profiling, electron microscopy for morphological assessment, and nanoparticle tracking analysis for particle size distribution), or tangential flow filtration combined with size-exclusion chromatography (SEC), along with standardized protocols to minimize technical variability while balancing yield and scalability for clinical applications. Subsequent research should focus on systematically evaluating the strengths and limitations of various EV heterogeneity identification methods, investigating which combination strategies can achieve more efficient, rapid, and specific identification of EV heterogeneity, and further exploring how to integrate identification methods with separation techniques to enhance separation efficiency while minimizing the impact of heterogeneity on research outcomes and clinical applications.

**Table 3:** Advanced technologies for addressing the heterogeneity of extracellular vesicles (EVs)

	Principle	Advantages	Limitations	Citations
Nanoparticle tracking analysis (NTA)	By analyzing the Brownian motion and light scattering characteristics of nanoparticles, it is possible to achieve multiparametric measurements, including particle size, concentration, and zeta potential	Characterize the concentration and particle size distribution of EVs. Suitable for the detection of low-concentration samples.	The inability to identify the nature of EVs, coupled with potential differences in particle size detection due to variations in laser wavelengths. Limited by the resolution capacity of the optical system, it is difficult to define a uniform minimum detection limit. The light scattering of large particles may affect the detection of small particles. The sensitivity of the instrument is constrained by its detection range, leading to questionable data authenticity when the particle size falls outside the optimal detectable range. A practical lower-size detection limit ranging from approximately 70 to 90 nm.	^[39-42,46-48]^
Dynamic light scattering (DLS)	By detecting the intensity fluctuations of scattered light when a laser illuminates the sample to measures the particle size distribution in solutions or suspensions	Determining the size distribution and integrity of EVs. Suitable for the detection of high-concentration and dispersed samples.	It is susceptible to dust and impurities, and the scattering signals produced by smaller particles or low-concentration particles may be overwhelmed by background noise, making accurate detection difficult. If the sample concentration is too low, there will not be enough scattered light for measurement. However, if the sample is too concentrated, multiple scattering will occur and the strong scattering signals from large particles will mask those from small particles, thereby leading to inaccurate measurement results.	^[70,86]^
Resistive pulse sensing (RPS)	By detecting the resistance changes caused when individual particles pass through micro-holes to measure the size and quantity of particles	High sensitivity and high resolution, suitable for particles of all sizes. In contrast to the inability of NTA, DLS, or imaging flow cytometry to provide traceable limits of detection (LOD), RPS method can offer definite LODs in both size and concentration.	Limited by the selection of micro-pore size, multiple pore diameters of micro-pores may be required to accommodate particles of different sizes. Measurement results may be affected by factors such as micro-pore blockage. Detection limits as small as approximately 50 nm in diameter.	^[45,70,87]^
Multi-angle light scattering (MALS)	Based on the principle of laser scattering to measure and analyze the molecular weight and molecular size distribution of substances	The measurement speed is fast, suitable for rapid analysis of a large number of samples. The equipment is relatively simple and the cost is low.	Only group statistical results can be provided, and detailed information of individual particles cannot be obtained. It is greatly affected by particle concentration, and either too high or too low concentration will affect the measurement results.	^[81,88]^
Raman spectroscopy (RS)	Based on the interaction between light and the chemical bonds within materials to provide information such as the chemical structure, phase and morphology, crystallinity, and molecular interactions of the sample	It can provide detailed chemical and molecular information and is suitable for the component analysis of EVs. Due to its high sensitivity and specificity, RS only requires a trace amount of sample for detection.	The instrument is expensive. The fluorescence signal is weak and is easily interfered by background light. In Fourier transform spectroscopy analysis, nonlinearity issues of the curve may arise, which require complex data processing and correction.	^[70,89,90]^
Scanning electron microscopy (SEM)	Acquiring information on the microstructure and composition of materials by focusing an electron beam to scan the surface of a sample	This device has high resolution and strong stereoscopic effect. SEM can not only be used to observe the morphology of samples, but also for component analysis. By equipping with devices such as EDS and EBSD, it can achieve microstructure morphology observation and crystal structure analysis.	SEM equipment is expensive, needs to operate in a vacuum environment, and has high requirements for the professional level of operators.	^[66,70,91]^
Transmission electron microscopy (TEM)	TEM generates images by irradiating samples with an electron beam in a high vacuum environment. By detecting the electrons that pass through the sample, an image can be formed.	Compared with SEM, which is used to observe the morphology and composition of the sample surface, TEM is mainly used to observe the internal structure of the sample and is suitable for studying nanoscale details such as atomic arrangement and crystal structure.	Samples usually need to be made into ultrathin sections with a thickness of about 50 nanometers. Electrons cannot easily penetrate parts with a thickness far greater than 200 nanometers. It can be used to prove the existence of EVs in the sample, but it seems not very suitable for proving the quality of the sample.	^[70,92,93]^
Cryo-electron microscopy (Cryo-EM)	By installing a sample cryogenic device on a conventional transmission electron microscope and cooling the samples to the temperature of liquid nitrogen (77 K), it is possible to observe temperature-sensitive samples such as proteins and biological sections.	By freezing the samples, the damage caused by the electron beam to the samples can be reduced, and the deformation of the samples can be minimized, thus obtaining a more authentic morphology of the samples.	Sample preparation is difficult and requires a high degree of uniformity in sample structure.	^[70,94]^
Scanning probe microscopy (SPM)	A physical probe or stylus contacts the surface of the sample and traverses it, scanning the curved profile and acquiring data to construct a topographic map of the sample’s surface.	High resolution (up to atomic level), three-dimensional imaging, suitable for various environments (such as liquids)	The imaging speed is relatively slow, and probe wear and contamination may affect the results.	^[70,95]^
Diffraction-limited fluorescence microscopy	Imaging is achieved within the visible light band based on the diffraction characteristics of light.	It can be used to observe the fine structures within cells through high-resolution imaging, which typically achieves a resolution of less than 250 nm.	The implementation of information technology is relatively complex, with a limited application scope and high requirements for signal processing.	^[70,83]^
Interferometric reflectance imaging sensing (IRIS)	High-resolution imaging, including single-particle interferometric reflectance imaging sensing and hybrid interferometric reflectance imaging-fluorescence microscopy, is achieved through interference reflection and fluorescence excitation.	It can perform detection at the single-particle level, with extremely high sensitivity, and is capable of detecting EVs at extremely low concentrations. Meanwhile, this technology can monitor the dynamic changes of EVs in real time, providing real-time information on the distribution and concentration of EVs.	This technical equipment is relatively expensive and not suitable for large-scale popularization. The operation is complex and its application range is limited.	^[70,77,96]^
Super-resolution microscopy (SRM)	Microscopy techniques that break through the diffraction limit of optical systems via physical or algorithmic approaches. According to the principles, they can be classified into different categories. Their development process is roughly as follows: stimulated emission depletion (STED), Single molecule localization microscopy (SMLM), minimal photon fluxes (MINFLUX), super-resolution optical fluctuation imaging (SOFI).	High resolution, detailed visualization, and wide applicability	The technology is complex, with high requirements for sample preparation and limitations in real-time imaging.	^[70,97]^
Bead-based flow cytometry	The technique of cell analysis involves using microspheres as markers and is performed by flow cytometry.	High sensitivity and specificity, non-invasive with multiple repeated detections, and multi-parameter analysis capabilities	The technology is complex and the cost is relatively high.	^[70,84,98]^
Single-EV flow cytometry	Precise classification and identification of individual EVs can be achieved through flow cytometry technology.	High sensitivity and specificity, multi-parameter detection capabilities, and high-throughput analysis	The technology is highly complex and challenging to standardize.	^[70,99]^
High-resolution flow cytometry	Based on the fundamental principles of flow cytometry, high-precision instruments and advanced detection technologies are employed to perform high-resolution analysis of cells or particles.	Multi-parameter detection, reduced sample usage and cost, rapid and accurate analysis, real-time dynamic monitoring, high throughput, high sensitivity, and high specificity.	The technology is complex, the cost is high, the sample processing requirements are stringent, and data analysis is intricate.	^[85,100]^
Genetic protein tagging	Through genetic engineering techniques, the DNA sequence encoding a specific tag is fused to the coding sequence of the target protein. Consequently, when the target protein is expressed, the tag is co-expressed, enabling the detection and purification of the target protein using specific methods (such as fluorescence microscopy, affinity chromatography, etc.).	High-efficiency expression and purification, as well as convenient detection	Potential functional interference, technical complexity, and high cost are notable challenges.	^[70,101]^
Mass spectrometry proteomics	By converting sample molecules into charged ions and subsequently separating and detecting them based on their mass-to-charge ratio (m/z), the molecular weight and structural information of the sample can be analyzed.	High throughput, high sensitivity and accuracy, as well as multi-functionality	The sample preparation process is cumbersome and time-consuming, data analysis is challenging, and the cost of equipment and reagents is relatively high.	^[70,102]^
Western blotting (WB)	Protein samples are first separated by electrophoresis and subsequently transferred to a solid-phase membrane. The target protein is then detected based on the principle of specific antigen-antibody binding.	It is a commonly used method for the quantitative and qualitative detection of target proteins and is readily recognized.	The expression level of the intracellular protein is low, while specific proteins exhibit significant differences between exosomes derived from normal cells and those from diseased cells.	^[70,103]^
Nucleic acid characterization	Identified through the detection of genetic material (such as DNA or RNA)	Suitable for bioinformatics analysis	Processing a large number of samples is time-consuming and may increase the risk of sample degradation. Meanwhile, such degradation can compromise the accuracy of result identification.	^[70,104]^
Enzyme-linked immunosorbent assay (ELISA)	Based on the principle of specific binding between antigen and antibody, signal conversion is achieved via an enzyme-substrate amplification system.	This method is characterized by high sensitivity and specificity, simple operation, and wide application.	Limited sensitivity and a potential risk of cross-reaction are notable drawbacks of this method.	^[105,106]^
Protein labelling of EVs	Protein labeling techniques, such as isotope labeling, fluorescence labeling, chemical labeling, and enzyme labeling, are employed to compare the relative abundance of proteins across different samples.	High quantitative accuracy, wide application range, and mature technology make this method highly advantageous for various analyses.	The operation is complex, the cost is high, and the requirements for sample processing are stringent.	^[70,71,107]^
Non-protein labelling of EVs	Non-protein labeling approaches rely on the comparison of mass spectrometry data from different samples to analyze changes in protein expression levels.	This method features simple operation, low cost, and a wide application range.	The quantitative accuracy is relatively low, and data processing is complex.	^[70,108]^
Droplet microfluidic	A technology that leverages the physicochemical properties and scale effects of multiphase microfluids to generate, manipulate, react, analyze, and screen microdroplets in microchannels.	This method is characterized by high-throughput processing capacity, low consumption of samples and reagents, high integration, and high flexibility.	The sequencing cost is high, the sequencing depth is limited, the technology is complex, and the equipment cost is substantial.	^[109,110]^
Biosensing	Based on biosensor technology, the target analyte is identified and its concentration is converted into an electrical signal for detection.	This method is characterized by high precision and sensitivity, wide application fields, multi-functionality, and the capability for real-time monitoring.	The method is associated with high cost and complex technology, and it is susceptible to external environmental influences, such as temperature and humidity, leading to poor stability of the sensor.	^[111,112]^
Single-vesicle technologies	It refers to the technology capable of detecting at the level of individual EVs.	This method is distinguished by high resolution and accuracy, as well as specific functional characterization.	Single vesicle technology demands highly specialized equipment and operational skills. Moreover, due to the large and complex volume of data generated, data processing and analysis necessitate advanced bioinformatics skills and considerable time investment. Additionally, maintaining consistency and reproducibility in large-scale research remains a significant challenge.	^[113-115]^

## Exosome-based therapy for OA

Exosomes, as one of the earlies characterized subpopulations, represent the most extensively investigated extracellular vesicular entities. Extensive empirical investigations have elucidated the modulatory capabilities of exosomes in diverse pathophysiological processes, with special emphasis on their functional roles in OA etiopathogenesis and therapeutic interventions^[116,117]^. This section systematically reviews exosomal subpopulations categorized by cellular derivation – specifically chondrocyte-derived, stem cell-derived, and heterogeneous exosomal populations – in the context of OA therapeutic applications (Table 4), with emphasis on their mechanistic roles in cartilaginous matrix regeneration, inflammatory cascade modulation, and articular homeostasis maintenance.

**Table 4:** Functional comparison of exosomes from different cellular origins in OA

Exosome source cell	Key bioactive molecules/cargo	Primary function in OA	Key signaling pathway/mechanism	Citations
Mesenchymal Stem Cells (MSCs)	miRNAs (e.g., miR-92a-3p, miR-140-5p, miR-100-5p); lncRNAs (e.g., TUC339, SNHG7); Proteins (e.g., MATN3)	Chondroprotective, Anti-inflammatory, and Pro-reparative	(1) Promotes chondrocyte proliferation and matrix synthesis. (2) Inhibits chondrocyte apoptosis, autophagy, and ferroptosis. (3) Modulates macrophage polarization towards M2 phenotype. (4) Regulates signaling pathways such as Wnt, mTOR, and PI3K/AKT	^[118-123]^
Chondrocytes	From Normal Cells (e.g., miR-95-5p); From OA Cells (e.g. Pro-inflammatory and pro-catabolic molecules)	Normal: Homeostasis maintenance, OA: Disease exacerbation	Normal: Promotes chondrogenesis and maintains matrix homeostasis, OA: Activates inflammasomes; promotes cartilage calcification and osteophyte formation	^[124-127]^
Fibroblast-like Synoviocytes (FLSs)	From Pathogenic FLSs: Pro-inflammatory cytokines and enzymes; From Engineered FLSs: miR-146a, SOD3	Pathogenic: Pro-inflammatory and pro-degradative; Engineered: Anti-inflammatory and chondroprotective	Pathogenic: Enhances macrophage glycolysis and induces chondrocyte apoptosis, Engineered: Inhibits the NF-κB signaling pathway and promotes M2 macrophage polarization	^[128-130]^
Macrophages	From M2-phenotype(miR-26b-5p), From M1-phenotype (miR-146b-5p)	M2-phenotype: Anti-inflammatory and chondroprotective, M1-phenotype: Pro-inflammatory	M2-phenotype: Inhibits TLR3 and COL10A1 expression; promotes lymphangiogenesis for inflammatory clearance, M1-phenotype: Amplifies the inflammatory cascade	^[131-133]^
Platelets	Growth factors, Cytokines, Pro-reparative molecules	Pro-reparative and Chondroprotective	(1) Enhances chondrocyte proliferation and migration. (2) Attenuates cartilage degenerative processes. (3) Improves subchondral bone microarchitecture.	^[134-136]^
Plants	Plant-derived bioactive molecules (e.g., SOX, ACAN from tomato)	Anti-inflammatory; Chondroprotective; and Accessible	(1) Upregulates the expression of chondrogenic marker genes. (2) Mitigates IL-1β-induced cartilage matrix degradation. (3) High production efficiency with low cytotoxicity	^[137,138]^

### Stem cell-derived exosomes

**Figure 2.**
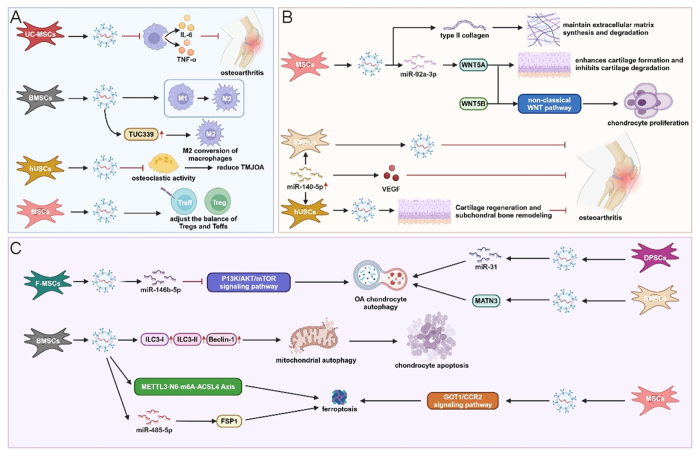
Mechanisms of delayed osteoarthritis (OA) progression mediated by exosomes derived from mesenchymal stem cells. MSC-derived exosomes alleviate arthritis through three primary mechanisms: anti-inflammatory effects (blue section, A), chondroprotection (yellow section, B), and metabolic regulation (pink section, C). Specifically, in terms of immune microenvironment modulation, exosomes secreted by umbilical cord-derived MSCs (UC-MSCs) inhibit macrophage secretion of IL-6 and TNF-α to mitigate OA. Exosomes derived from bone marrow MSCs (BMSCs) promote M1-to-M2 macrophage polarization and upregulate TUC339, which enhances M2 polarization. Exosomes secreted by human umbilical cord stromal cells (hUSCs) suppress osteoclast activity to reduce temporomandibular joint OA (TMJOA). Additionally, MSC-derived exosomes regulate the balance between regulatory T cells (Tregs) and effector T cells (Teffs). Regarding chondrocyte protection, MSC-derived exosomes increase type II collagen expression to maintain extracellular matrix homeostasis. Moreover, miR-92a-3p-enriched exosomes inhibit cartilage degradation by promoting cartilage formation via the WNT5A pathway. In terms of anti-oxidative stress and metabolic imbalance, miR-146b-5p in exosomes secreted by fetal MSCs (F-MSCs) induces autophagy in OA chondrocytes by inhibiting the PI3K/AKT/mTOR signaling pathway. Similarly, exosomes secreted by dental pulp stem cells (DPSCs) and synovial membrane MSCs (SMSCs) promote autophagy through miR-31 and MATN3, respectively. BMSC-derived exosomes also enhance autophagy by upregulating ILC3-I, ILC3-II, and beclin-1, thereby inducing mitochondrial autophagy. Furthermore, BMSC-derived exosomes induce ferroptosis via the METTL3-m6A-ACSL4 and miR-485-5p/FSP1 axes. Finally, MSC-derived exosomes trigger ferroptosis through the GOT1/CCR2 signaling pathway. Created with Biorender.

Stem cells, defined by their exceptional self-renewal capacity and multipotent differentiation potential represent key cellular components in regenerative therapeutic applications. Their reparative efficacy in OA pathophysiology is mediated through multiple mechanistic pathways, including chondrogenic differentiation, immunomodulatory functions, and extracellular vesicle-mediated paracrine signaling^[117]^. A substantial body of empirical investigations has provided compelling evidence that stem cell-derived exosomal populations possess significant potential to attenuate osteoarthritic disease progression and demonstrate superior therapeutic efficacy compared to exosomes derived from alternative cellular sources. Zhu *et al* performed a comparative analysis of the therapeutic potential of exosomes derived from induced pluripotent stem cell-derived mesenchymal stem cells (iMSC-Exos) versus synovial membrane mesenchymal stem cell-derived exosomes (SMMSC-Exos) using *in vitro* human chondrocyte models. Subsequently, they assessed their efficacy in a collagenase-induced murine OA model. Their experimental results indicated that iMSC-Exos exhibited significantly greater therapeutic potency than SMMSC-Exos^[139]^ (Fig. 2).

#### The protection of chondrocytes by stem cell-derived exosomes

In recent years, exosomal populations derived from MSCs have become a predominant focus of investigation for promoting cartilaginous tissue regeneration and inhibiting matrix degradation processes^[140–143]^. Mao *et al* reported increased expression levels of exosomal miR-92a-3p in an MSC-chondrogenic differentiation model, whereas exosomal fractions derived from osteoarthritic chondrocytes exhibited significantly reduced expression levels. Experimental administration of MSC-derived miR-92a-3p-enriched exosomes (MSC-miR-92a-3p-Exos) or MSC-derived anti-miR-92a-3p-loaded exosomes (MSC-anti-miR-92a-3p-Exos) to MSCs and primary human chondrocytes demonstrated that miR-92a-3p specifically targets WNT5A, thereby enhancing chondrogenic differentiation and alleviating matrix degradation processes^[118]^. Although exosomal WNT5A and WNT5B stimulate chondrocyte proliferative activity through non-canonical WNT signaling pathways, they may also exert modulatory effects on extracellular matrix synthesis. Tao *et al* demonstrated that synovial MSC-derived exosomal populations overexpressing miR-140-5p (SMSC-miR-140-5p-Exos) effectively counteract these effects, thereby promoting chondrocyte proliferation and reparative processes. These exosomes also exhibited superior osteoarthritis-preventive efficacy in a rodent experimental model^[119]^. Liu *et al* further demonstrated that exosomal populations derived from miR-140-5p-overexpressing human urine-derived stem cells (hUSCs) exhibit an enhanced capacity to promote cartilaginous regeneration and subchondral bone remodeling compared with standard hUSC-derived exosomes (hUSCs-Exos). Vascular endothelial growth factor (VEGF) was identified as a direct target of miR-140, contributing to the enhancement of extracellular matrix secretion. These findings were validated in a rat experimental model^[144]^. Wang *et al* utilized interleukin-1β-treated primary murine chondrocytes and a destabilization of the medial meniscus (DMM)-induced osteoarthritis model to demonstrate that exosomal populations derived from embryonic stem cell-induced mesenchymal stem cells enhance type II collagen synthesis and significantly alleviate cartilaginous destruction and matrix degradation processes, thereby preserving the balance between extracellular matrix synthesis and degradative pathways^[145]^. Among diverse stem cell sources, cartilage-derived stem/progenitor cells (CSPCs) exhibit superior efficacy in cartilaginous tissue repair, and their corresponding exosomal derivatives display comparable regenerative properties. In a subacute osteoarthritic rat model, Chen *et al* demonstrated that exosomal fractions derived from CSPCs effectively promote cartilaginous regeneration, with cyclin-dependent kinase 9 identified as a key molecular mediator^[143]^.

#### The modulation of the immune microenvironment by stem cell-derived exosomes

Dysregulated immune responses within specific tissue microenvironments serve as a key pathological mechanism driving disease progression^[146]^. Zhang *et al* demonstrated *in vitro* that exosomes derived from bone marrow mesenchymal stem cells (BMSC-Exos) facilitate the phenotypic transition of synovial macrophages from the pro-inflammatory M1 subtype to the anti-inflammatory M2 subtype, thereby alleviating cartilage damage mediated by M1 macrophages^[147]^. Shen *et al* further demonstrated that BMSC-Exos mediate this immunomodulatory effect by upregulating TUC339 expression, thereby promoting macrophage polarization toward the M2 phenotype^[120]^. Similarly, exosomes derived from human urine stem cells (USC-Exos) have been shown to inhibit osteoclastic activity in subchondral bone, thereby alleviating temporomandibular joint OA (TMJ-OA) pathology^[148]^. Elevated levels of inflammatory cytokines, such as IL-6 and TNF-α, exacerbate chondrocyte injury and matrix degradation^[149]^. Klyucherev *et al* reported that exosomes derived from umbilical cord mesenchymal stem cells (UC-MSC-Exos) significantly suppress IL-6 and TNF-α secretion in human monocyte-derived macrophages, thereby alleviating OA-associated inflammation^[150]^. Additionally, T-cell dysregulation, particularly the imbalances between regulatory T cells (Tregs) and effector T cells (Teffs), plays a pivotal role in synovial inflammation in OA patients. Exosomes derived from MSCs (MSC-Exos) restore Treg/Teff homeostasis, thereby providing therapeutic potential for mitigating OA symptoms^[151]^.

#### The role of stem cell-derived exosomes in alleviating oxidative stress and restoring metabolic balance

Chondrocyte autophagy is a phylogenetically conserved self-degradative cellular process that plays a critical role in promoting chondrogenic differentiation and extracellular matrix biosynthesis. Dysregulation of autophagic flux has been identified as a prevalent pathophysiological mechanism underlying osteoarthritic disease progression^[121]^. Wu *et al* demonstrated that infrapatellar fat pad MSCs-derived exosomal populations, which are enriched in miR-100-5p, enhance autophagic processes in osteoarthritic chondrocytes by suppressing the mammalian target of rapamycin (mTOR) signaling pathway, thereby exerting chondroprotective effects and alleviating gait abnormalities^[121]^. Similarly, fucoidan-preconditioned MSCs-derived exosomal preparations promote chondrocyte autophagic flux through miR-146b-5p-mediated inhibition of the phosphatidylinositol 3-kinase/protein kinase B/mammalian target of rapamycin (PI3K/AKT/mTOR) signaling cascade^[152]^. Ji *et al* further elucidated that exosomal miR-182-5p attenuates osteoarthritic pathology by promoting tumor necrosis factor alpha-induced protein 8 (TNFAIP8) overexpression, which subsequently activates autophagy-related protein 3-microtubule-associated protein 1A/1B-light chain 3 (ATG3-LC3)-mediated autophagic processes^[153]^. Additionally, dental pulp stem cell-derived exosomal fractions (DPSC-Exos) enriched in miR-31 enhance chondrocyte autophagic mechanisms by inhibiting mTOR signaling, thereby ameliorating osteoarthritic pathophysiology^[154]^. Moreover, in a murine OA model, synovial MSC-derived exosomal populations (SMSC-Exos) carrying matrilin-3 (MATN3) effectively restore autophagic flux and delay extracellular matrix degradation processes^[155]^.

Mitochondrial dysfunction is strongly correlated with osteoarthritic disease progression through diverse mechanistic pathways, reflecting a well-established pathophysiological relationship^[156,157]^. Shen *et al* identified that GATD3A deficiency enhances the interaction between Sirt3 and MDH2, thereby exacerbating mitochondrial dysfunction and accelerating senescence in fibroblast-like synoviocytes, which in turn drives OA progression^[158]^. Under mitochondrial stress, cells activate mitophagy to maintain homeostasis. A study demonstrated that BMSC-derived exosomes (BMSC-Exos) upregulate the expression of autophagy-related proteins LC3-II/LC3-I and Beclin-1 in chondrocytes, thereby enhancing mitophagy and suppressing apoptosis^[159]^. Further expanding on this paradigm, another group isolated mitochondrial EVs (mitoEVs) from MSCs and demonstrated their ability to deliver functional mitochondria into antimycin-induced chondrocytes with compromised mitochondrial activity, thereby proposing mitoEVs as a promising therapeutic vector for OA^[160]^. Ma *et al* developed a multifunctional exosome-based system (Gel@Exo(modAtf5)) by integrating gene delivery vectors, MSC-derived exosomes, and thermosensitive hydrogels for the overexpression of activating transcription factor 5 (ATF5), thereby effectively alleviating mitochondrial dysfunction and OA pathology^[161]^. Furthermore, Chen *et al* developed an advanced biomimetic scaffold system for mesenchymal stem cell exosome delivery using desktop stereolithographic additive manufacturing technology to fabricate a three-dimensional extracellular matrix/gelatin methacryloyl/exosome (3D-printed ECM/GelMA/exosome) composite scaffold with radially aligned microchannels. This bioengineered scaffold system exhibits significant efficacy in enhancing chondrocyte mitochondrial function, promoting cellular migration, and inducing synovial macrophage polarization toward the anti-inflammatory M2 phenotype, thereby substantially augmenting cartilaginous tissue regeneration in preclinical animal models^[162]^.

Chondrocyte ferroptosis constitutes a crucial mechanistic pathway in osteoarthritic disease progression. Empirical studies have shown that MSC-derived exosomal populations (MSC-Exos) exert chondroprotective effects against osteoarthritic pathophysiology by suppressing ferroptotic cell death mechanisms through modulation of the glutamic-oxaloacetic transaminase 1/C-C motif chemokine receptor 2 (GOT1/CCR2) signaling axis^[122]^. BMSC-Exos similarly suppress ferroptotic processes by inhibiting the methyltransferase-like 3-N6-methyladenosine-acyl-CoA synthetase long-chain family member 4 (METTL3-m6A-ACSL4) molecular pathway^[163]^. Wang *et al* elucidated that BMSC-Exo fractions enriched in small nucleolar RNA host gene 7 (SNHG7-rich BMSC-Exos) effectively inhibit ferroptotic cell death mechanisms by modulating the microRNA-485-5p/ferroptosis suppressor protein 1 (miR-485-5p/FSP1) signaling axis^[123]^. In the context of osteoarthritic therapeutic interventions using exosomal preparations, strategies to address suboptimal vesicular yields have been explored^[164]^. Wu *et al* elucidated that tumor necrosis factor-alpha (TNF-α) preconditioning significantly enhances the secretory capacity of infrapatellar fat pad-derived MSC exosomal populations via activation of the phosphatidylinositol 3-kinase/protein kinase B (PI3K/AKT) signaling cascade^[164]^. Furthermore, recent technological advancements have enabled the development of MSC-derived nanovesicular constructs (MSC-NVs), which, compared to endogenously produced mesenchymal stem cell exosomal populations, exhibit significantly enhanced production efficiency and superior functional properties related to cartilaginous tissue regeneration and anti-inflammatory efficacy. When incorporated into methacrylated gelatin (GelMA) hydrogel delivery systems, these engineered nanovesicular formulations enable temporally controlled release kinetics, thereby optimizing therapeutic efficacy^[165]^.

#### Differences in therapeutic effects presented by exosomes from different stem cell sources

The cargo of exosomes, including microRNAs and proteins, differs based on the cell source, culture conditions, and disease state. This heterogeneity poses a significant challenge to standardization, which remains a critical barrier to clinical translation. A comparative analysis of studies utilizing protein secretomes from adipose (AD), bone marrow (BM), placenta (PL), and Wharton’s jelly (WJ)-derived human MSCs revealed that each MSC secretome profile exhibits distinct characteristics based on its source. Despite sharing common features, such as promoting cell migration and regulating programmed cell death negatively, variations in the composition of the secretome were observed across different sources. When compared with adult MSCs, fetal MSCs secreted a more diverse range of proteins, thereby demonstrating stronger functional properties and greater relevance. The secretome of fetal-derived MSCs, such as PL and WJ, exhibited a more diverse composition compared to that of AD and BM-derived MSCs^[166]^. When comparing and evaluating the regenerative potential of MSC-derived EVs (cEVs) and platelet-derived EVs (pEVs) in an OA cartilage rat model, Forteza-Genestra MA *et al* found that knee joints treated with pEVs exhibited superior subchondral bone integrity in CT-analyzed parameters compared to those treated with cEVs or in the OA group. This indicates that pEVs may serve as a promising regenerative treatment for OA, as they not only exert regenerative effects on osteoarthritic cartilage but also demonstrate greater efficacy than cEVs, particularly in female rats^[167]^. After reviewing the clinical trials of MSCs for OA treatment, Zhang X *et al* concluded that placenta-derived MSCs and UC-MSCs possess two major advantages over BM-MSCs and AD-MSCs: (i) they exhibit comparable regenerative potential while maintaining low immunogenicity, and (ii) they can be obtained through non-invasive methods, making them more accessible^[168]^. In addition to cell source, the culture environment of stem cells also influences their therapeutic effects as well as those of the extracellular vesicles they secrete^[169]^. With adipose-derived MSCs (ADSCs) cultured under standard conditions, in the presence of high levels of IFNγ, under low-level inflammatory conditions mimicking OA synovial fluid (SF), and in OA-SF, Ragni E *et al* found that different culturing conditions influenced the size and release of EVs and modulated the overall landscape of ADSC-derived EV-miRNAs^[170]^. Similarly, to better understand the pathogenesis and identify potential treatments, Klymiuk MC *et al* harvested EVs from equine ADSCs that were cultivated and pretreated under various conditions: interleukin-1β exposure, shock wave treatment, chondrogenic differentiation, chondrogenic differentiation under hypoxia, or senescence. They found that different types of stimulation induced significant changes in the expression levels of several miRNAs^[171]^. In the research on detecting biomarkers for pre-screening Alzheimer’s disease, Cano A *et al* found that CSF p-tau^181^ levels were correlated with plasma EVs in early-onset mild cognitive impairment (EOMCI) patients with established amyloidosis, indicating that plasma EVs change as Alzheimer’s disease progresses^[172]^. After systematically reviewing the role of EVs in the pathogenesis, diagnosis, and treatment of OA, Lin J *et al* concluded that EVs from different cell sources not only contribute to the progression of OA but also serve as effective tools for its diagnosis and treatment^[173]^. Disease is a dynamic developmental process. Exosomes produced by different cell types in a disease microenvironment also exhibit distinct characteristics. Further research is warranted to elucidate the damaging and therapeutic effects of exosomes derived from various cell sources, culture conditions, and disease states, thereby enabling efficient and reproducible case-specific utilization of exosomes.

### Cartilage-derived exosomes

**Figure 3.**
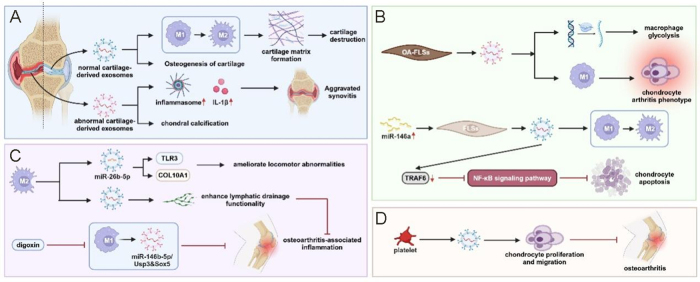
Mechanisms of delayed osteoarthritis (OA) progression by exosomes derived from cartilage and other sources. (A) Exosomes secreted by normal cartilage promote M1-to-M2 macrophage polarization and enhance cartilage formation. In contrast, exosomes produced by osteoarthritic chondrocytes stimulate the generation of inflammatory vesicles and factors that exacerbate arthritis and contribute to cartilage calcification. (B) Exosomes secreted by fibroblast-like synoviocytes overexpressing miR-146a also facilitate M1-to-M2 polarization. Additionally, these exosomes downregulate TRAF6, inhibit the NF-κB signaling pathway, and prevent chondrocyte apoptosis. Exosomes released by osteoarthritic fibroblast-like synoviocytes regulate glycolysis through transcriptional mechanisms and mediate a model of chondrocyte arthritis induced by M1 macrophages. (C) MiR-26a-5p-enriched exosomes secreted by M2 macrophages alleviate motility abnormalities and enhance lymphatic drainage via TLR3 and COL10A1 signaling. Administration of digoxin suppresses M1 secretion of miR-146b-5p/Usp3 and Sox5, thereby mitigating arthritis. (D) Platelet-derived exosomes promote chondrocyte proliferation and migration while alleviating arthritis. Created with Biorender.

Chondrocytes represent the sole resident cellular population within articular cartilage and play a pivotal role in secreting exosomal cargo, which constitutes a critical mediator for regulating the cartilaginous microenvironment^[174]^. Mao *et al* demonstrated that miR-95-5p expression is significantly downregulated in osteoarthritic chondrocyte-derived exosomes compared to those isolated from non-pathological cartilage. Administration of primary chondrocyte-derived exosomes with engineered overexpression of miR-95-5p (AC‑miR-95-5p-Exos) significantly enhanced mesenchymal stem cell chondrogenic differentiation and extracellular matrix synthesis, whereas co-cultivation with exosomes transfected with miR-95-5p antisense inhibitory sequences (AC-anti-miR-95-5p-Exos) suppressed chondrogenesis, upregulated histone deacetylase 2/8 (HDAC2/8) expression, and reduced cartilaginous matrix production. Mechanistically, AC-miR-95-5p-Exos regulate cartilage developmental processes and homeostatic maintenance by inhibiting interactions with reporter gene vectors containing the HDAC2/8 3′ untranslated region^[124]^. Normal chondrocyte-derived exosomal populations exhibit the capacity to attenuate osteoarthritic disease progression through immunomodulatory mechanisms. Sang *et al* developed a thermosensitive, injectable hydrogel formulation by in situ cross-linking of Pluronic F-127 and hyaluronic acid. Incorporation of primary chondrocyte-derived exosomes followed by intra-articular administration promoted cartilage regeneration by facilitating M1-to-M2 macrophage phenotypic polarization and enhancing cartilaginous matrix synthesis, thereby significantly mitigating degenerative pathology^[125]^. Given the predominant role of chondrocytes in cartilage homeostasis, normal primary chondrocyte-derived exosomal populations are likely to attenuate osteoarthritic disease progression. Conversely, Ni *et al* demonstrated that exosome-like vesicular structures derived from osteoarthritic chondrocytes activate inflammasome complexes, thereby stimulating macrophage-mediated production of mature interleukin-1β and exacerbating osteoarthritis-associated synovial inflammation^[126]^. Liu *et al* elucidated that aberrant biomechanical loading parameters enhance the secretion of chondrocyte-derived exosomes. In TMJ-OA, these vesicular populations promote cartilage calcification processes^[175]^. Shi *et al* observed that tensile mechanical stress induces alterations in microRNA expression profiles within rat condylar chondrocyte-derived exosomes, thereby promoting osteogenic differentiation. These exosomal populations also facilitate condylar cartilage ossification^[127]^. Collectively, these findings indicate that normal chondrocyte-derived exosomes promote chondrogenic processes, whereas pathologically altered exosomal populations exhibit detrimental effects. Whether exogenous exosomal administration alone has sufficient capacity to modulate joint cartilage differentiation trajectories remains a critical question requiring further investigation (Fig. 3).

### Other exosomes

Diverse cellular populations contribute to the exosomal secretome that modulates the osseous microenvironmental milieu. Exosomal populations originating from fibroblast-like synoviocytes, tissue-resident and circulating macrophages, activated platelets, and phyto-derived sources demonstrate therapeutic potential in osteoarthritic pathophysiology. Nevertheless, the paucity of comprehensive investigative studies in this domain necessitates extensive further empirical exploration to elucidate their mechanistic contributions and therapeutic efficacy parameters (Fig. 3).

#### Exosomes derived from fibroblast-like synoviocytes

Fibroblast-like synoviocytes (FLSs), the predominant cell type in synovial fluid, contribute to OA progression through EV-mediated secretion and inflammatory signaling^[176,177]^. Liu *et al* demonstrated that osteoarthritic fibroblast-like synoviocyte-derived exosomal fractions (FLS-Exos) promote macrophage polarization toward the pro-inflammatory M1 phenotype, thereby inducing osteoarthritic phenotypic characteristics in chondrocyte populations. Furthermore, FLS-Exos significantly enhances macrophage glycolytic metabolic pathways by upregulating glycolytic enzymatic genes through hypoxia-inducible factor 1-alpha (HIF-1α)-mediated transcriptional regulation to meet elevated energetic demands during inflammatory processes. These mechanistic insights indicate that FLS-Exos-induced macrophage functional dysregulation may serve as a potential therapeutic target for osteoarthritic disease management^[128]^.

Conversely, Wang *et al* demonstrated that FLS-Exos overexpressing microRNA-146a effectively facilitate the phenotypic conversion of macrophages from the pro-inflammatory M1 subtype to the anti-inflammatory M2 subtype. Furthermore, these exosomes downregulate TNF receptor-associated factor 6 (TRAF6) expression and inhibit nuclear factor-kappa B (NF-κB) signaling pathways, ultimately mitigating osteoarthritis-related structural deterioration of articular cartilage and chondrocyte apoptosis. These findings underscore the substantial therapeutic potential of microRNA-146a as a molecular mediator for OA protection^[129]^. Cao *et al* utilized single-cell transcriptomic sequencing methodologies, identified superoxide dismutase 3 (SOD3) as a potential regulatory target within synovial tissues that significantly augments chondrocyte antioxidant enzymatic capacity. The enrichment of fibroblast-like synoviocyte-derived exosomal preparations containing SOD3 (S-EXO), followed by their incorporation into hydrogel microsphere-based delivery systems for controlled release, enables precise and efficient delivery of these exosomes to articular cartilaginous tissues^[130]^.

#### Macrophage-derived exosomes

Extensive investigative studies have established a robust correlation between macrophage functionality and the pathophysiology of OA^[178]^. Liu *et al* utilized flow cytometric quantification methodologies to enumerate circulating monocyte populations based on differential expression of CD86 (an M1 phenotypic marker) and CD163 (an M2 phenotypic marker), demonstrated that osteoarthritic patients exhibit a statistically significant increase in the M1/M2 phenotypic ratio. This ratio positively correlates with radiographic Kellgren-Lawrence grading classifications^[179]^. Qian *et al* demonstrated that exosomal microRNA-26b-5p derived from M2 macrophages confers chondroprotective effects by specifically inhibiting the expression of Toll-like receptor 3 (TLR3) and collagen type X alpha 1 chain (COL10A1). This mechanism alleviates locomotor abnormalities in murine osteoarthritic models, highlighting its substantial therapeutic potential^[131]^. Jia *et al* demonstrated that digoxin attenuates osteoarthritis-associated inflammatory processes by inhibitory modulation of the microRNA-146b-5p/ubiquitin-specific peptidase 3 & SRY-box transcription factor 5 (miR-146b-5p/Usp3/Sox5) molecular axis in M1 macrophage-derived exosomal populations. Consequently, therapeutic strategies aimed at reducing M1 phenotypic polarization or promoting M2 phenotypic conversion represent promising interventional approaches for osteoarthritic disease management^[132]^. Empirical investigations have revealed that exosomal fractions derived from M2 macrophages (M2Exos) effectively promote the proliferation of synovial lymphatic vasculature. A hydrogel-based delivery system engineered for controlled release kinetics of M2Exos significantly enhanced lymphatic drainage functionality and effectively mitigated the progression of osteoarthritic disease in both in vitro experimental systems and in vivo animal models^[133]^. Furthermore, specific investigative approaches have employed exosomal populations derived from macrophages as pharmaceutical delivery vehicles to inhibit transforming growth factor-beta (TGF-β) signaling activity within subchondral bone microenvironments for osteoarthritic therapeutic applications. Jing *et al* engineered vesicular constructs derived from macrophages incorporating the peptide DSS6 and encapsulating Galunisertib, thereby enabling the therapeutic agent to bypass immunological clearance mechanisms, traverse biological barrier interfaces, and achieve targeted localization within subchondral osseous structures^[180]^.

#### Platelet-derived exosomes

Platelet-rich plasma (PRP) therapeutic interventions have shown efficacy in alleviating postoperative nociception in recipients of total knee arthroplasty and improving clinical symptomatology associated with osteoarthritic pathophysiology^[181,182]^. Empirical investigations have demonstrated that platelet-derived EV populations (pEVs) exhibit substantial regenerative and reparative potential across diverse tissue microenvironments^[134]^. Xu *et al* demonstrated that pEVs significantly enhance the proliferative and migratory capacities of chondrocyte *in vitro*, mitigate cartilaginous degenerative processes, improve subchondral bone microarchitectural parameters, and effectively delay the progression of osteoarthritic disease *in vivo*^[135]^. Forteza-Genestra *et al* established an *ex vivo* osteoarthritic experimental model using human cartilage explant systems and demonstrated that, compared to UC-MSC-EVs preparations, therapeutic administration of pEVs resulted in significantly increased deoxyribonucleic acid content and enhanced collagenous matrix deposition. This underscores the substantial therapeutic potential of pEVs in acellular regenerative medicine approaches for osteoarthritic disease management^[136]^.

#### Tumor-derived exosomes

Tumor-derived exosomal populations have received significant investigative attention within the scientific community. Jiang *et al* demonstrated that exosomal fractions derived from the MB49 bladder carcinoma cell line are internalized by macrophages and subsequently induce polarization toward the anti-inflammatory M2 phenotype via the downregulation of phosphatase and tensin homolog (PTEN) expression and concurrent activation of protein kinase B/signal transducer and activator of transcription 3/6 (AKT/STAT3/6) signaling cascades^[183]^. This molecular mechanism may facilitate a reduction in the M1/M2 macrophage phenotypic ratio, thereby potentially mitigating osteoarthritic symptomatology. However, it is important to note that tumor-derived EVs have been extensively documented to promote neoplastic progression and play critical functional roles in tumorigenic initiation and metastatic dissemination^[184,185]^.

#### Plant-derived exosomes

In recent years, exosomal populations derived from diverse plant sources have received considerable attention for their potential applications in articular injury therapeutics. Phytogenic exosomal preparations exhibit enhanced accessibility and production efficiency. For instance, tomato-derived exosomal vesicular fractions (TELV) effectively upregulate the expression of chondrogenic marker genes, specifically SRY-box transcription factor (SOX) and aggrecan (ACAN), without inducing significant cytotoxic effects^[137]^. Garlic-derived exosomal populations (GDEs) co-cultured with interleukin-1β-stimulated chondrocytes could significantly mitigate interleukin-1β-induced cartilaginous matrix degradation. In addition, intra-articular administration of GDEs resulted in significant improvement in locomotor parameters in murine osteoarthritic models^[138]^.

## Microvesicles as therapeutic agents for OA

Microvesicles (MVs), alternatively referred to microparticles (MPs), have been extensively investigated for their distinctive molecular cargo composition and functional properties compared to exosomal populations across diverse physiological and pathophysiological contexts^[186,187]^. Sheng *et al* demonstrated that osteoclast-derived MVs, unlike exosomes or apoptotic bodies promote synovial homeostasis via dual tissue-reparative (chondrogenic and osteogenic) and anti-inflammatory functions, thereby establishing MVs as a mechanistically distinct therapeutic modality for OA^[188]^. Yu *et al* suggested that microvesicular fractions derived from BMSCs (BMSC-MVs) exerted superior efficacy compared to exosomal preparations in enhancing chondrocyte mitochondrial functional parameters. Moreover, intra-articular administration of these microvesicular populations effectively mitigated osteoarthritic disease progression in rodent experimental models^[189]^. Oxidative stress, caused by an overproduction of reactive oxygen species (ROS), is strongly associated with mitochondrial dysfunction in chondrocytes^[190]^. Guillén *et al* demonstrated that MVs derived from ADSCs possess a superior antioxidant capacity compared to exosomes, likely due to their higher enrichment in the antioxidant enzyme peroxiredoxin-6 (Prdx6)^[191]^. AbuBakr *et al* using a monosodium iodoacetate-induced temporomandibular joint osteoarthritis (MIA-induced TMJ-OA) rat model, stratified experimental animals into three distinct groups: untreated control, BMSC-MVs, and platelet-rich plasma (PRP)-treated. Both intervention groups demonstrated significant improvements in articular disc tissue morphology along with concurrent reductions in the expression of proinflammatory mediators, specifically interleukin-1β (IL-1β), tumor necrosis factor-alpha (TNF-α), nuclear factor-kappa B (NF-κB), matrix metalloproteinase-13 (MMP-13), and matrix metalloproteinase-3 (MMP-3). Notably, BMSC-MVs administration resulted in superior therapeutic outcomes compared to PRP treatment^[192]^.

**Figure 4.**
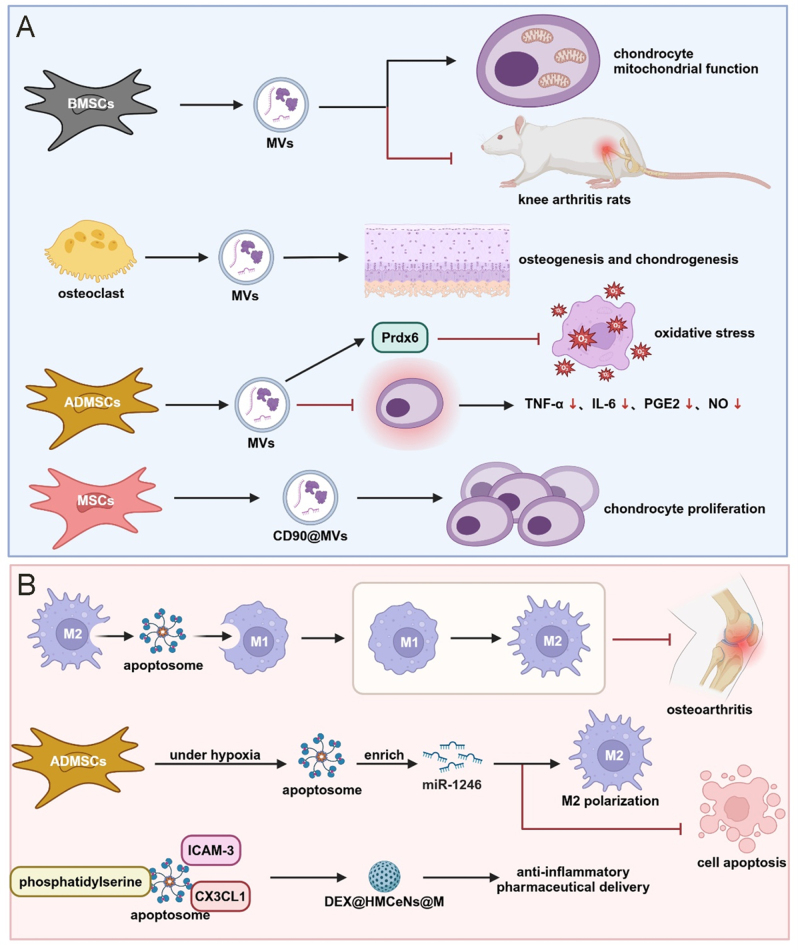
Mechanisms of microvesicles and apoptotic bodies in delaying osteoarthritis (OA) progression. The mechanism by which microvesicles alleviate OA are highlighted in the blue section, while those of apoptotic vesicles are shown in the red section. (A) Regarding microvesicles, BMSC-derived microvesicles modulate chondrocyte mitochondrial function and effectively treat animal models of osteoarthritis. Osteoclast-derived microvesicles enhance both osteogenesis and chondrogenesis. AD-MSC-secreted microvesicles target Prdx6 to inhibit oxidative stress and downregulate pro-inflammatory cytokines such as TNF-α, IL-6, PGE2, and NO. CD90-positive microvesicles (CD90@MVs) promote chondrocyte proliferation. (B) In the apoptotic vesicle fraction, apoptotic vesicles facilitate M1-to-M2 macrophage polarization. Hypoxia-induced AD-MSC-secreted apoptotic vesicles enriched in miR-1246 further promote M2 polarization and inhibit apoptosis. Additionally, ICAM-3, CX3CL1, and phosphatidylserine encapsulated in DEX@HMCeNs@M can deliver anti-inflammatory drugs. Created with Biorender.

MVs and exosomes exhibit distinct characteristics. However, they share certain similarities in their anti-inflammatory mechanisms, such as the inhibition of TNF-α cascades^[193]^. Cosenza *et al* elucidated that microvesicular and exosomal preparations exhibit comparable efficacy in osteoarthritic pathophysiology by restoring chondrocyte metabolic homeostasis, inhibiting programmed cell death mechanisms, and conferring chondroprotective effects, thereby demonstrating significant therapeutic potential in a collagenase-induced murine osteoarthritis experimental model^[194]^. Tofiño-Vian *et al* isolated adipose-derived mesenchymal stem cells (AD-MSCs) from lipoaspirate specimens and subsequently extracted microvesicular and exosomal fractions from the conditioned culture medium. Their findings demonstrated that both vesicular subtypes exhibit comparable efficacy in suppressing interleukin-1β-induced chondrocyte inflammatory responses, significantly mitigating the production of tumor necrosis factor-alpha (TNF-α), interleukin-6 (IL-6), prostaglandin E2 (PGE2), and nitric oxide^[195]^. MVs, due to their high cargo-loading capacity, exhibit significant potential as drug delivery vehicles. Building on this potential, researchers engineered CD90 + MSCs-derived MVs (CD90@MVs) as a drug carrier, showcasing robust therapeutic efficacy in a rabbit OA model. CD90@MVs not only preserved the pro-chondrogenic properties of their parental cells but also enhanced drug delivery efficiency, highlighting their dual functionality in OA therapy^[196]^ (Fig. 4).

## Apoptotic bodies as potential therapeutic agents for OA

Apoptotic bodies represent specialized membranous structures generated during the terminal stages of programmed cell death (apoptosis), characterized by distinct morphological features and encapsulated within a phospholipid bilayer envelope. In contrast to other extracellular vesicular subpopulations, apoptotic bodies exhibit restricted cellular tropism predominantly targeting professional phagocytic cells (e.g., macrophages, dendritic cells) and neighboring non-phagocytic cellular entities. These vesicular structures play critical functional roles in apoptotic cellular debris clearance mechanisms and intercellular signaling transduction, thereby demonstrating considerable therapeutic potential in diverse biomedical applications, including tissue regenerative engineering, pharmaceutical delivery systems, and immunomodulatory therapeutic interventions^[197]^. Apoptotic bodies, which generally range from 1 to 5 μm in diameter, display size-dependent functional heterogeneity. Recent studies have highlighted a distinct subclass of smaller apoptotic vesicles (0.1–1 μm) that stimulate cellular proliferation and differentiation. This contrasts with the inhibitory effects observed for their larger counterparts (1–5 μm)^[198]^. This size-dependent functional divergence underscores their potential for targeted OA therapy. Current research on apoptotic bodies in OA treatment predominantly centers on their anti-inflammatory properties, which are primarily mediated through macrophage phagocytosis and subsequent immunomodulation. Qin *et al* performed a comprehensive investigation of M2 macrophage-derived apoptotic bodies (M2-ABs) within a murine OA model, revealing that pro-inflammatory M1 macrophages exhibit selective internalization of M2-ABs within a 24-hour temporal window. This internalization process promotes phenotypic conversion toward the immunoregulatory M2 phenotype, thereby mitigating M1-mediated inflammatory microenvironments and consequently alleviating osteoarthritic pathological severity. Mechanistically, this process is associated with miR-12-5p^[199]^. Ding *et al* demonstrated that hypoxic preconditioning of ADSCs leads to the production of apoptotic bodies with augmented cartilaginous reparative capacity compared to those generated under normoxic conditions. Comprehensive transcriptomic and proteomic analyses identified substantial enrichment of microRNA-1246 in these hypoxia-conditioned vesicular structures, which exhibited enhanced efficacy in suppressing chondrocyte apoptotic cascades and driving macrophage polarization toward the anti-inflammatory M2 phenotype^[200]^.

Apoptotic bodies have shown considerable potential in biomedical engineering and drug delivery applications, attributed to their unique biophysical characteristics and excellent biocompatibility^[201,202]^. Apoptotic bodies express specific molecular markers, such as fractalkine (CX3CL1) and intercellular adhesion molecule-3 (ICAM-3), which mediate macrophage chemotactic recruitment, along with externalized phosphatidylserine residues that promote phagocytic internalization. The role of apoptotic bodies in OA pathogenesis extends significantly beyond macrophage polarization alone. While their involvement in macrophage polarization is well-documented, the therapeutic potential of apoptotic bodies as drug delivery vehicles, as highlighted by studies such as Chen *et al*^[201]^, represents a particularly exciting and under-explored avenue in OA treatment. Lever-aging these intrinsic biological properties, Chen *et al* developed an osteoarthritis-targeted anti-inflammatory therapeutic platform based on apoptotic chondrocyte membrane-coated hollow mesoporous cerium oxide nanospheres functioning as ROS scavengers. The resultant dexamethasone-loaded hollow mesoporous cerium oxide nanospheres coated with chondrocyte membranes (DEX@HMCeNs@M) delivery system, presenting native “eat-me” signals, effectively mimics cartilaginous apoptotic body structures to facilitate targeted, spatiotemporally controlled anti-inflammatory pharmaceutical delivery^[203]^. Chen *et al*’s study on dexamethasone-loaded apoptotic body mimetic nanospheres exemplifies the growing potential of leveraging apoptotic bodies’ inherent biological advantages for targeted OA therapy. While apoptotic bodies play multifaceted roles in OA pathogenesis and resolution – ranging from immunomodulation beyond macrophages to chondroprotection and efferocytosis – their utilization as sophisticated, naturally inspired drug delivery vehicles represents a paradigm shift in OA therapeutic strategies. Studies such as Chen *et al*’s work on dexamethasone-loaded nanospheres highlight the immense potential of leveraging apoptotic bodies’ unique biological properties, including immune-evasive stealth, inherent targeting to inflammatory cells, biocompatibility, and intrinsic therapeutic cargo, to address the delivery challenges that have hindered many OA therapies. This bio-inspired approach merits significantly expanded research focus due to its potential for targeted, sustained, synergistic, and potentially disease-modifying treatments for OA. Moving forward, optimizing fabrication techniques, enhancing scalability, improving loading efficiency, and investigating long-term safety will be critical translational steps toward clinical implementation.

While all EV subpopulations have been investigated, current research predominantly centers on exosomes, with relatively fewer studies on MVs and apoptotic bodies. This phenomenon can be attributed to the research history, technical limitations, and the inherent biological characteristics of different vesicle subtypes. (I) There are challenges in separation and purification methods. Compared with exosomes, which exhibit relatively uniform size and density, microvesicles and apoptotic bodies display a broader size distribution. Traditional separation techniques are predominantly optimized for exosomes. The characteristic tetraspanin protein family of exosomes facilitates their capture via immunoadsorption, whereas specific markers for microvesicles and apoptotic bodies remain largely unstandardized. Moreover, the membrane compositions of microvesicles and apoptotic bodies closely resemble those of their parent cells, complicating efforts to achieve consistent separation. (II) The biological and historical evolution should be focused further. Exosomes rapidly garnered significant attention from researchers due to their early identification as nucleic acid carriers involved in gene regulation and were subsequently regarded as promising tools for disease diagnosis and treatment. In contrast, microvesicles and apoptotic bodies were long overlooked and dismissed as mere cellular debris. Only recently have researchers begun to recognize their distinct biological functions and therapeutic potential. (III) The immunomodulation functions among vesicle subtypes are different and should be systematically compared. Exosomes are widely considered a relatively safe therapeutic option due to their low immunogenicity. While microvesicles possess strong cargo-loading capacity, their procoagulant and pro-inflammatory properties introduce uncertainties in clinical applications. Apoptotic bodies, originating from dying or dead cells, may trigger autoimmunity, necessitating stricter safety evaluations and thereby potentially slowing research progress. Each subpopulation exhibits distinct advantages and limitations. Exosomes, characterized by their broad cellular sources, low immunogenicity, and small size, demonstrate high safety profiles in clinical applications when derived from autologous or stem cell sources. However, large-scale production and purification remain technically challenging. In contrast, MVs exhibit superior cargo-loading capacity due to their larger size but pose risks of pro-inflammatory effects and thrombogenicity, along with technical difficulties in separating them from apoptotic bodies during purification. Apoptotic bodies, despite their high yield, face similar purification challenges and may trigger autoimmune responses due to residual nucleic acids (Fig. 4). The lack of satisfactory preparation strategies represents a common challenge for all three types of EVs. In addressing the challenges of standardization and quality control, engineering modification may emerge as a key future development trend for EVs.

## Administration routes for EVs

Nonpharmacologic interventions, pharmacotherapeutic approaches, and surgical methodologies represent the primary therapeutic modalities for OA management. Surgical interventions, however, may lead to significant postoperative complications, such as aseptic implant loosening following arthroplastic procedures^[204]^. Conventional pharmacological regimens often induce multisystemic adverse effects, thereby redirecting research focus toward minimally invasive cellular therapeutic approaches. Compared with cellular therapeutic interventions, extracellular vesicular preparations – acting as critical mediators of intercellular signaling transduction – exhibit several advantageous features, such as simplified isolation procedures, improved storage stability, scalable dosing, and reduced immunogenicity^[205]^. Although the mechanistic pathways by which EVs mediate osteoarthritic disease attenuation have been comprehensively elucidated in preceding sections, the development of efficient targeted delivery systems for therapeutic EV constructs remains a paramount challenge in contemporary translational research efforts.

Intravenous administration is a widely adopted delivery modality owing to its favorable pharmacokinetic profile, which is characterized by rapid absorption and swift onset of therapeutic efficacy. Despite the reduced thrombogenic potential of EVs compared to cellular therapeutic entities due to their nanoscale dimensions, residual thromboembolic risk remains, necessitating strict control of infusion dosage parameters and administration rate^[206]^. Yang *et al* demonstrated that high-concentration (≥1 μg/g body mass) intravenous administration of UC-MSC-EVs induced the formation of multiple pulmonary microthrombi in murine experimental models, leading to acute mortality^[207]^. Moreover, extracellular vesicular preparations exhibit accelerated *in vivo* clearance kinetics. To address this limitation, biocompatible polymeric matrices, such as hydrogel delivery systems, have been developed to significantly enhance vesicular residence time within target tissues^[208]^. Oral administration, distinguished by superior patient compliance, has undergone extensive investigative evaluation. Researchers have successfully isolated and purified tea leaf-derived exosome-like nanoparticulate structures (TLNTs) and, through comparative murine experimental analyses of intravenous versus oral delivery routes, demonstrated that intravenous administration induced significantly greater hepatorenal cytotoxicity and immunological activation compared to enteral delivery modalities^[209]^. Nevertheless, the acidic microenvironmental conditions within the gastrointestinal tract, along with proteolytic and lipolytic enzymatic activity, significantly compromise the efficacy of oral extracellular vesicle delivery. Empirical investigations have shown that EV populations derived from intestinal epithelial cells and mammalian milk exhibit promising potential for enhancing oral bioavailability^[210]^. Building upon this mechanistic foundation, Liu *et al* demonstrated that milk-derived EVs effectively attenuate osteoarthritic disease progression by augmenting cartilaginous tissue thickness, enhancing extracellular matrix biosynthetic processes, and concurrently reducing catabolic enzymatic expression profiles^[211]^.

**Figure 5.**
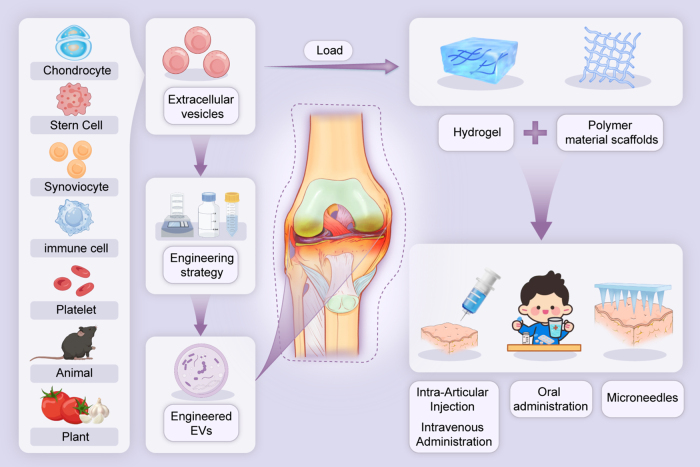
EV sources, engineering strategies, and delivery routes. EVs derived from a variety of cellular sources, can be functionally enhanced through advanced bioengineering strategies such as encapsulation within hydrogels or surface modification. Current delivery approaches encompass the following: (1) Intravenous injection, which enables rapid systemic distribution but carries the risk of thrombosis due to EV aggregation; (2) Intra-articular injection, which minimizes systemic toxicity yet requires invasive administration by trained clinicians; (3) Oral delivery, which offers high patient compliance but suffers from low bioavailability due to degradation in the gastrointestinal tract; (4) Microneedle systems, which combine minimally invasive delivery with the potential for patient self-administration, though optimizing payload retention remains a challenge.

Compared with systemic delivery modalities, intra-articular administration provides superior localized therapeutic efficacy by directly delivering EVs to affected articular structures. This approach facilitates enhanced local concentration while concurrently mitigating systemic adverse effects^[212]^. Gupta *et al* conducted a systematic evaluation of the safety profile and therapeutic efficacy of intra-articularly administered cell-free stem cell-derived extracts (CCM) in a cohort of 20 patients with grade II/III OA, revealing favorable safety outcomes and significant symptomatic amelioration^[213]^. However, potential adverse sequelae may occur, for instance, intra-articular hyaluronic acid administration often induces, injection-site nociception and inflammatory edema^[214]^. Given the inflammatory microenvironment characteristic of osteoarthritic joints, enhanced phagocytic activity and accelerated synovial fluid turnover kinetics may substantially diminish the therapeutic efficacy of EVs, necessitating repetitive administration protocols that could potentially exacerbate iatrogenic adverse reactions. Contemporary investigative paradigms have increasingly focused on microneedle-based delivery technologies, which, upon transdermal insertion, generate microchannels that effectively minimize extravasation and immunological interference while providing minimally invasive, sustained-release pharmacokinetic profiles^[215]^. Recent technological advancements have enabled the development of a microneedle-based polydopamine-coated exosome-loaded microneedle (PDA@ExoMN) delivery platform. This platform synergistically leverages the transdermal penetration capabilities of microneedle arrays and the ROS scavenging properties of exosomes to confer chondroprotective effects and effectively attenuate osteoarthritic disease progression^[216]^ (Fig. 5).

## Clinical application of EVs in OA

As EVs are increasingly recognized as a novel therapeutic approach for the treatment of OA, it is essential to conduct clinical trials to verify the effectiveness of EVs in OA. Multiple preclinical and clinical studies have demonstrated the effectiveness of EVs for damaged cartilage, paving the way for the advancement of clinical research^[217]^. Gupta A *et al* conducted a preliminary non-randomized, open-label, multi-center feasibility and safety study to evaluate the safety and efficacy of cell-free stem cell-derived extract (CCM) from human progenitor endothelial stem cells (hPESCs) in patients suffering from grade II/III knee OA. The outcomes of this initial study on novel CCM will lay the foundation for a larger, randomized, placebo-controlled, multi-center clinical trial investigating intraarticular CCM for symptomatic knee OA^[213]^. However, the results have not yet been released. After assessing the efficacy and safety of sEVs derived from umbilical cord MSCs (UC-MSC-sEV) with in vitro studies and OA mouse models, Figueroa-Valdés AI *et al* conducted the first-in-human application of cGMP-sEV therapy for OA and observed that intra-articularadministration of UC-MSC-sEV resulted in notable and long-lasting improvements in pain and disability^[218]^. Another clinical study of Wang Y *et al* demonstrated that human UC-MSC-derived exosomes effectively alleviate inflammation and promote cartilage regeneration in OA^[219]^. On the contrary, the clinical trial conducted by Bolandnazar NS *et al* demonstrated that a single intra-articular injection of placental MSC-derived EVs (5 cc, 7 × 10^9 particles/cc) is safe. However, it does not improve clinical symptoms or MRI findings in knee OAbeyond placebo effects^[220]^. More detailed clinical studies are presented in Table 5. Overall, clinical research on the role of Evs in OA is still in its infancy, and most studies remain in the early stages (phase I). Although the research findings remain controversial, most researchers agree that EVs exert a beneficial effect on OA. With the advancement of exosome extraction techniques and the clarification of their heterogeneity, there is a need for more large-scale studies to evaluate the role of EVs in OA, thereby promoting more in-depth clinical trials.

**Table 5:** Key clinical trials involving extracellular vesicles (EVs) for OA

Clinical trial number and registration date	Origin and key characteristics of EVs	Source and characteristics of cases	Experimental types and detailing phase	Outcomes	Limitations	Citations
NCT04719793 (registered on 22 January 2021)	Umbilical cord-derived Wharton’s jelly (including growth factors (GFs), cytokines (CKs), hyaluronic acid (HA), and EVs	Twelve patients with Kellgren grade II/III OA who meet the inclusion and exclusion criteria will be recruited for this non-randomized, open label, multi-center, prospective study. The study will be conducted at two sites within the USA, and the patients will be followed for 1 year, with an expected duration of 15 months	A non-randomized, open-label, multi-center, prospective study (detailing phase is not clearly marked. It is inferred to be phase I based on the experiment content)	Not yet reported	Not yet reported	^[221]^
NCT05060107 (Last Update Posted 28 September 2021)	Intra-articular knee injection of exosomes (3-5 x 10e11 particles) derived from allogeneic mesenchymal stromal cells. Single dose.	10 knee OA Kellgren-Lawrence grade II to III patients were enrolled in this trial and the follow-up will be up to 12 months.	A prospective study (phase I)	No results yet posted	Not yet reported	^[222]^
NCT04711304 (registered on 15 January 2021)	Umbilical cord (UC)-derived Wharton’s jelly (WJ) are compared to hyaluronic acid (HA, control) and saline (placebo control) in patients	A total of 168 participants with grade II or III knee OA on the Kellgren grade scale will be recruited across 53 sites in the USA with 56 participants in each arm and followed for 1 year post-injection.	A randomized, controlled, single-blind, multi-center, prospective study (detailing phase is not clearly marked. It is inferred to be phase I based on the experiment content)	Not yet reported	The present investigation was designed as single-blinded rather than double-blinded. Another limitation of the study is the utilization of only one HA formulation.	^[223]^
NCT04971798 (registered on 21 July 2021)	A novel cell-free stem cell-derived extract (CCM) from human progenitor endothelial stem cells (hPESCs) with presence of growth factors (GFs), cytokines (CKs), and EVs	The study will be conducted at up to 2 sites within the USA, and the 12 participants with Kellgren grade II or III knee OA will be followed for 24 months.	A non-randomized, open-label, multi-center, prospective study (detailing phase is not clearly marked. It is inferred to be phase I based on the experiment content)	Not yet reported	Not yet reported	^[213]^
IRCT20210423051054N1 (approved on 11 May 2022)	A single intra-articular injection of placental mesenchymal stromal cells-derived extracellular vesicles (5 cc, 7 × 10^9^ particles/cc)	62 knees (31 patients suffering from bilateral knee osteoarthritis with Kellgren grade 2 or 3) were enrolled in this study. There were 31 knees as intervention and 31 knees as control.	A randomized, triple-blind, placebo-controlled clinical trial (detailing phase is not clearly marked. It is inferred to be phase I based on the experiment content)	No statistically significant difference was detected between the two groups in clinical outcomes (including VAS, WOMAC, and Lequesne scores) before treatment and 2 and 6 months after treatment. Also, no statistically significant difference was detected between the two groups in MRI findings before treatment and 6 months after treatment. No systemic complications or severe local reactions occurred in the patients.	The same patient’s one knee was used as a control, while the contralateral knee was subjected to the intervention experiment. This limitation might have influenced the overall scores in the WOMAC and Lequesne indices. Additionally, the relatively limited sample size may have contributed to the negative outcomes.	^[220]^
None	The cGMP-sEV (2 × 10^10^ ± 0.5 × 10^10^ particles) packed into a syringe was kept at 4 °C until IA administration in the patient.	A 56 year-old woman with a limited gait range of 40 m and Kellgren-Lawrence stage II OA evident on radiography was recruited for the IA injection of sEV.	First-in-human sEV administration in osteoarthritic knee (phase I)	Following IA administration, the patient exhibited marked and sustained improvements in both pain and disability. Clinical assessments, including VAS and WOMAC index scores, demonstrated significant improvements at 6 months that were maintained through the 12-month follow-up.	The sample size is small.	^[218]^
NCT06431152 (Last Update Posted 29 May 2024)	UC-MSC-sEV	Three cohorts are planned with four subjects in each cohort receiving a three-fold escalating dose of 2 × 10^9^ ± 0.5 × 10^9^ total particles, 6 × 10^9^ ± 0.5 × 10^9^ total particles, or 2 × 10^10^ ± 0.5 × 10^10^ total particles. 12 patients with Kellgren-Lawrence grade II-III knee OA (Rosenberg view x-ray).	A pilot dose-escalation study (phase I)	No results yet posted	Not yet reported	^[224]^
MR-13-24-017929 (registered 11 February 2023)	Exosomes derived from human umbilical cord mesenchymal stem cells (hUC-MSCs-Exos)	41 patients with 45 knee joints were assigned to three treatment groups: low dose group (3 × 10^11^ exos, *n* = 15), middle dose group (4 × 10^11^ exos, *n* = 15), high dose group (5 × 10^11^ exos, *n* = 15). All patients had Kellgren-Lawrence grade 2 or 3 OA in the knee joints and were followed up for 9 months.	A randomized, double-blind, dose-escalation clinical trial (phase I)	HUC-MSC-derived exosomes effectively alleviate inflammation and promote cartilage regeneration in osteoarthritis, demonstrating safety and efficacy in both preclinical and clinical studies.	(1) Small sample sizes; (2) The effects of hUC-MSCs-Exos in severe OA patients remain unclear; (3) All patients included in this study were below K-L grade 4; (4) MRI re-examinations at the 9-month follow-up.	^[219]^
NCT06466850 (Last Update Posted 20 June 2024)	Allogenic mesenchymal stem cells derived exosomes	20 patients with moderate to severe osteoarthritis (Kellgren-Lawrence grade 2 or 3 in both knees) and accompanied by mild to moderate pain and swelling were included.	A pilot study (detailing phase is not clearly marked. It is inferred to be phase I based on the experiment content)	No results posted	Not yet reported	^[225]^
NCT06937528 (Last Update Posted 22 April 2025)	Extracellular vesicles (EV)	50 patients with severe KOA stage III or IV by Laurance & Kellgren staging as judged by Posterioranterior Xray of the knee joint.	A prospective study (phase I)	No results posted	Not yet reported	^[226]^

## Conclusions and perspectives

OA significantly impairs the quality of life in middle-aged and elderly populations. Non-pharmacological interventions, including exercise and weight management, demonstrate greater efficacy in delaying OA progression compared to late-stage pharmacological or surgical treatments, yet traditional therapeutic approaches remain constrained by limited effectiveness. Recent investigations into intercellular communication mechanisms have highlighted EVs as pivotal mediators. Due to their diverse cargo, EVs play a crucial role in regulating inflammation, repairing cartilage damage, and promoting tissue regeneration in OA. In contrast to cell-based therapies, which encounter challenges such as immune rejection, teratoma formation risks, and ethical concerns, EV-based acellular therapies offer a promising alternative with enhanced safety and feasibility.

In the isolation of EVs, ultracentrifugation (UC) remains the most widely adopted method and is frequently regarded as the gold standard. Nevertheless, a growing body of evidence indicates that size-exclusion chromatography (SEC) may yield superior outcomes in certain contexts. Despite these advancements, no single isolation technique is devoid of limitations, and the integration of multiple methods is currently considered more practical for achieving optimal results. For EV characterization, it is recommended to employ five categories of marker proteins. Analogous to the isolation process, a combination of characterization approaches is essential to fulfill the stringent requirements for comprehensive and reliable analysis. EVs isolated from the synovial fluid and blood of OA patients display distinct surface markers compared to those derived from healthy individuals. This characteristic can facilitate clinical OA staging and the targeted isolation of pathogenic EVs for therapeutic applications. EVs are generally classified into three subpopulations, with exosomes (Exos) being the most extensively studied. Virtually all cell types are capable of secreting Exos, among which MSC-Exos have been the most thoroughly investigated. MSC-Exos carry a diverse array of miRNAs and modulate multiple signaling pathways, including the Wnt and mTOR pathways, thereby promoting chondrocyte proliferation while mitigating inflammation and senescence in OA. Despite their small size and strong tissue penetration, Exos encounter challenges such as low yield and difficulties in preparation. Microvesicles (MVs), which are larger and possess a higher cargo capacity, demonstrate therapeutic effects comparable to those of Exos in OA treatment but differ in specific aspects, such as the superior ability of Exos to enhance mitochondrial function in chondrocytes and their enhanced targeting capabilities. Apoptotic bodies, being the largest in diameter, can carry organelles and effectively clear inflammation. However, their limited sources and potential to induce damage to normal cells restrict their broader application. OA is a continuously progressive disease that exhibits distinct characteristics at different stages. EVs from different sources play varying roles at different stages of the disease. Likewise, EVs derived from different sources and isolated at different stages may exhibit differing therapeutic effects on OA. Therefore, stage-specific treatment strategies are necessary, and it is crucial to identify EVs suitable for each stage. Further research in these areas is warranted to enhance our understanding and refine therapeutic approaches.

Natural EVs can be directly utilized for OA treatment or engineered as drug carriers to minimize immune rejection and enhance therapeutic efficacy. Biomedical engineering techniques can further optimize EV targeting capabilities. Various administration routes are available for EV delivery: (1) intravenous injection rapidly elevates blood concentration but carries the risk of thrombosis; (2) oral administration offers improved patient compliance, with milk-derived EVs showing potential, although their localized therapeutic effects require enhancement; (3) intra-articular injection, commonly employed in OA treatment, concentrates EVs at the target site while reducing systemic side effects, yet repeated injections pose infection risks; (4) microneedles, an emerging delivery system, induce minimal skin damage; (5) EVs integrated with biomaterials such as hydrogels have demonstrated promising therapeutic outcomes in OA. However, several challenges persist in the application of EVs for OA treatment: (1) The absence of standardized protocols for the characterization, isolation, and purification of EV subpopulations impedes large-scale production. (2) The precise mechanisms underlying the therapeutic effects of EVs in OA remain incompletely elucidated, and the limited number of clinical trials necessitates further exploration of their safety and efficacy. (3) A suitable long-term delivery method and an optimized carrier design strategy are required to enhance bioavailability and facilitate clinical translation. Despite these challenges, the integration of technological advancements and interdisciplinary collaboration offers promise for overcoming these obstacles and advancing EV-based OA therapies.

## Data Availability

Not applicable.
